# Transcriptomic and metabolomic responses of maize under conventional and biodegradable microplastic stress

**DOI:** 10.1002/imo2.48

**Published:** 2024-12-28

**Authors:** Yuanze Sun, Jingxi Zang, Siyuan Xie, Mochen Wu, Jianguo Tao, Tanveer M. Adyel, Xinyu Du, Si Li, Jie Wang

**Affiliations:** ^1^ Beijing Key Laboratory of Farmland Soil Pollution Prevention and Remediation, College of Resources and Environmental Sciences China Agricultural University Beijing China; ^2^ Centre for Nature Positive Solutions, Biosciences and Food Technology Discipline, School of Science RMIT University Melbourne Victoria Australia; ^3^ Collage of Maine Ecology and Environment Shanghai Ocean University Shanghai China

**Keywords:** biodegradable microplastics, chemical defense, conventional microplastics, DOM, maize, multi‐omics, rhizosphere microorganisms

## Abstract

The increasing accumulation of microplastics in agricultural soils potentially threatens crop safety and quality. However, studies regarding the molecular mechanisms underlying the effects of conventional and biodegradable microplastics on plant growth remain limited. Herein, we estimated the effects of biodegradable polybutylene adipate terephthalate, poly (butylene succinate), polylactic acid, and conventional non‐biodegradable polyethylene and polystyrene microplastics (at a concentration of 1% [w/w]) on the growth and physiological performance of maize (*Zea mays L*.). In addition, we studied the molecular mechanisms underlying the effects of these microplastics on maize. Exposure to microplastics induced the production of antioxidant enzymes and antioxidants at varying levels in the maize. While the maize antioxidant systems were induced against biodegradable microplastic exposure, maize photosynthesis was relatively more important for conventional microplastic treatments. Additionally, metabolomics and transcriptomic analyses revealed that the pathways of secondary metabolite biosynthesis, photosynthesis, energy metabolism, and carbohydrate metabolism were regulated by biodegradable and conventional microplastics. Specifically, microplastics induced the plant hormone signal transduction and mitogen‐activated protein kinase signaling pathways. Our results further indicated that microplastics could impact the plant through changing the soil environmental variables or altering the soil microbial communities. This study provides a molecular‐scale perspective on the responses of crops to microplastic contamination, and these findings will contribute to the ecological risk assessment of biodegradable and conventional microplastics.

## Highlights

- Microplastics triggered antioxidant defense system, especially under biodegradable microplastic exposure.
- Microplastic exposure induced the plant hormone signal transduction and mitogen‐activated protein kinase signaling pathways.
- Effects of microplastics on plants resulted from changing the soil environmental variables or altering the soil microbial communities.

## Graphical abstract

This study revealed that microplastics activated the antioxidant defense system and enhanced maize photosynthesis. Metabolomic and transcriptomic analyses showed that biodegradable and conventional microplastics regulated pathways related to secondary metabolite biosynthesis and carbohydrate metabolism. Belowground changes played a key role in mediating maize responses to microplastic stress.

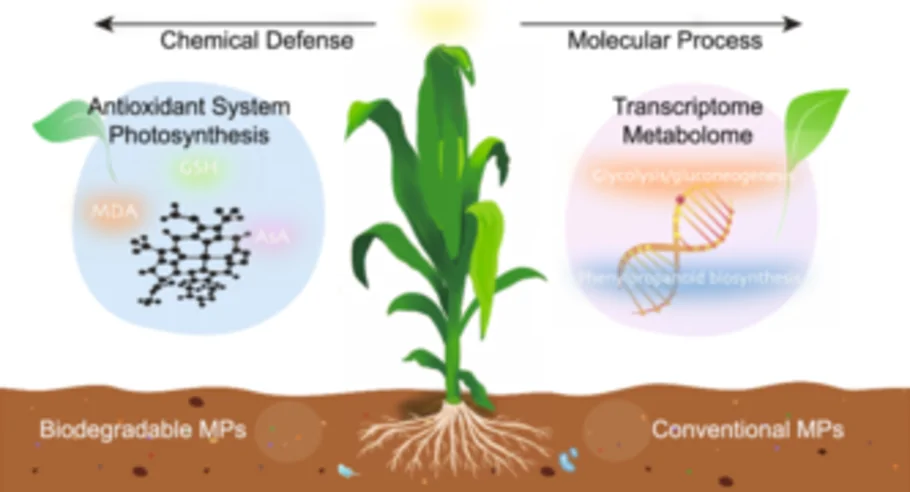

## 1 INTRODUCTION

With the increasing production and application of plastics, microplastics (diameter < 5 mm), as emerging contaminants, are ubiquitously detected in various environments, for example, marine water [1], freshwater [2], atmosphere [3], soil ecosystems [4], and even in the glaciers and above the planetary boundary layer [5, 6]. Due to the application of sewage sludge and compost, wastewater irrigation, degradation of plastic mulching, and atmospheric deposition, croplands have been presumed to be the major reservoirs of microplastics, and microplastics in polluted soils could reach up to 6.7% of soil weight in extreme cases [7, 8, 9]. Currently, microplastics have been validated to exhibit various adverse consequences on the abiotic and biotic components in agroecosystems, including the modification of soil physicochemical properties, the spread of potentially pathogenic microbes, the interference in microbial functions, the impacts on plant growth, and even human exposure via the food chain [10, 11, 12, 13]. The threats to long‐term food security raised crucial concerns in the field of microplastic contamination. To resolve the intractable microplastic pollution problem, biodegradable plastics are developed and promoted as the ideal substitutes for traditionally petroleum‐based plastics. Several biodegradable polymers, including polybutylene adipate terephthalate (PBAT), polylactic acid (PLA), and poly butylene succinate (PBS), have been applied in agricultural activities, food industries, and medical treatments [14]. It has been estimated that the global production of biodegradable plastics will reach approximately 6.3 million tonnes in 2027 [15]. Theoretically, microorganisms can eventually mineralize biodegradable polymers to carbon dioxide and water. However, recent studies indicated that the degradation of biodegradable plastics will take years or even longer under natural conditions, and these plastics would also undergo fragmentation into small particles during their degradation [16]. Additionally, owing to their intrinsic properties, biodegradable plastics would disintegrate into microplastics more rapidly than conventional plastics, which may result in more substantial negative effects on soil ecosystems [17]. Therefore, it is vital to understand the effects of both conventional and biodegradable microplastics on agroecosystems for more sustainable use of plastic products.

Recent studies have explored the effects of microplastic residuals on the crop performance. Yang et al. reported that PBAT and polyethylene (PE) microplastics significantly reduced the height and dry weight of rice and caused oxidative stress to the shoots and roots [18]. Brown et al. found that biobased microplastic poly(3‐hydroxybutyrate‐co‐3‐hydroxyvalerate) (PHBV) caused a dose‐dependent reduction of maize growth [19]. However, researchers observed the opposite result, that microplastics (PLA and polystyrene (PS)) increased the growth of peanuts [20]. This suggests that, apparently, there is no consensus in the scientific community on the phytotoxic effects of microplastics. The potential reason may be that microplastics lead to nonvisible and subtle effects on crops at the physiological, biochemical, and molecular levels. Hence, only evaluating the visible phytotoxicity is rather inadequate. Understanding the changes in regulatory mechanisms of gene expression in crops following microplastic exposure is crucial. Furthermore, previous studies suggested that changes in the composition of entire soil communities could lead to altered plant characteristics, e.g., plant biomass, physiology, genetics, and metabolism [21, 22, 23]. How microplastic‐associated changes in soil will influence the plant performance is less well known.

Maize (*Zea mays L*.) is the most widely grown grain globally and is used as a staple food; the grain remains an important source of income for farmers in developing countries [24]. Hence, this study investigated the primary growth index, antioxidant activities, and photosynthetic performance of maize upon microplastic exposure. The biobased microplastics, including PBAT, PBS, and PLA, and the petroleum‐based microplastics, including PE and PS, were used because of their wide application [25, 26]. To elucidate the underlying molecular mechanism of the biological responses of maize to microplastics, integrated transcriptomics and metabolomics studies were conducted. Additionally, to estimate whether the below‐ground changes have far‐stretching consequences for above‐ground maize performance under the exposure of microplastics, the analyses of microbial community composition (determined with 16S ribosomal RNA gene sequencing) and the fingerprint of dissolved organic matter (DOM) (determined with three‐dimensional excitation‐emission matrix fluorescence spectroscopy) in the rhizosphere soil were carried out. Our findings will provide a comprehensive and deep understanding of the effects of different polymer microplastics on crops and, therefore, help the sustainability of agricultural practice.

## 2 RESULTS AND DISCUSSION

### Microplastics triggered antioxidant defense system and improved photosynthesis

**FIGURE 1:**
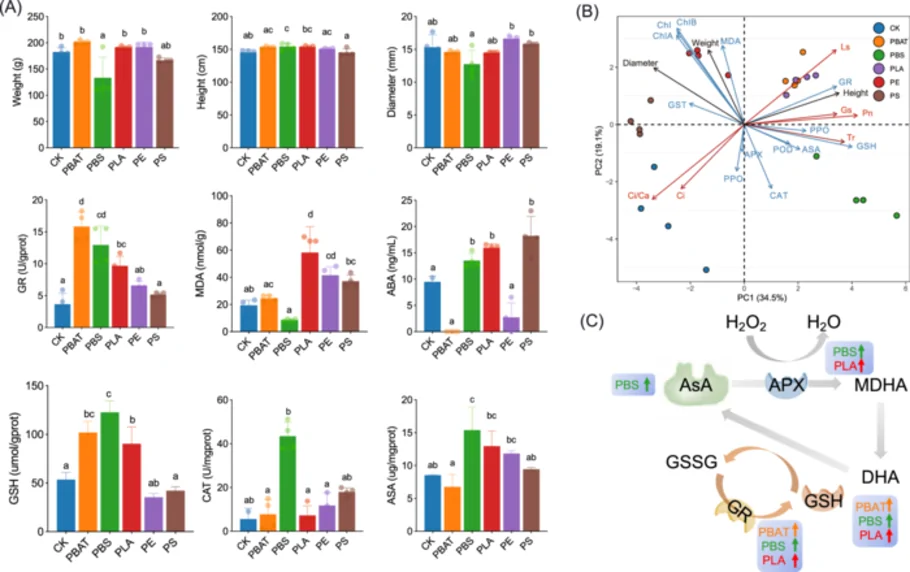
Responses of the antioxidant defense system and photosynthesis of maize to microplastic. Fresh weight, height, stem diameter, activities of glutathione reductase (GR), glutathione (GSH), glutathione S‐transferase (CAT), and ascorbic acid (ASA), and contents of malondialdehyde (MDA) and abscisic acid (ABA) in different treatments (A). Different letters denoted significant differences at 0.05 probability levels. Vertical bar represents standard error ± SE among four replicates. Principal component analysis (PCA) of maize growth parameters under different microplastic treatments (B). Schematic representation of ascorbate‐glutathione (ASA‐GSH) cycle under biodegradable microplastic treatments (C).

The phenotypical changes that occurred in maize were determined. After 60 days of exposure to microplastics, only PBS microplastics significantly decreased the fresh mass and diameter of the maize by 27% and 17%, respectively, whereas other microplastics showed limited effects on the growth of maize (Figure 1A). In regard to the antioxidative responses of maize to microplastics, the levels of lipid peroxidation products (malondialdehyde (MDA)), antioxidant molecules (glutathione (GSH) and ascorbic acid (ASA)), activities of antioxidant enzymes (glutathione reductase (GR) and catalase (CAT)), and nonenzymatic antioxidants (abscisic acid (ABA)) were induced to varying levels. Specifically, all three biodegradable microplastics increased the GR activities by 2.64–4.33 folds, whereas the CAT activities were only induced by PBS (7.80 folds). Additionally, PBS and PLA microplastics statistically increased the concentrations of GSH, ASA, and ABA. The MDA contents were induced when maize was treated with PE and PS, potentially suggesting oxidative damage. In comparison with the control, the conventional PE and PS microplastics increased the chlorophyll contents (Figure S3). For instance, the total concentration of chlorophyll increased by 46% and 42% after PE and PS treatment, respectively. However, biodegradable microplastics did not significantly impact on chlorophyll content. We also analyzed the effects of microplastics on maize photosynthetic parameters (Table S3). PBAT, PBS, PLA, and PE microplastics enhanced photosynthetic performance as demonstrated by the significant increase in the values of photosynthetic rate (Pn), stomatal conductance (Gs), intercellular carbon dioxide (Ci), transpiration rate (Tr), and stomatal limitation (Ls); Leaf intrinsic water use efficiency (LWUEint) and leaf instantaneous water use efficiency (LWUEins) were also enhanced. However, no significant changes were observed in response to PS treatments. To further visualize the different phenotypical responses to microplastics, principal component analysis (PCA) with all observations was conducted (Figure 1B). The results indicated that the first two principal components accounted for 51.3% of the total variances (PC1 and PC2 explained 34.5% and 19.1%, respectively). Additionally, the conventional and biodegradable microplastic treatments were separated along PC1, indicating that the effects of microplastics on maize physiology were distinct between the biobased and petroleum‐based polymers. The contents of chlorophyll and MDA were the most important variables for PE and PS treatments, whereas photosynthetic parameters, GR, and GSH were strongly associated with PBAT, PBS, and PLA treatment.

We further analyzed the relationship between the maize growth, antioxidative system, and photosynthetic performance. The growth characteristics of maize were significantly correlated with antioxidant enzyme (CAT), antioxidant molecules (GSH and ASA), chlorophyll, and photosynthesis performance (Tr, Ci, and Pn) (Figure S4). These results potentially indicated that maize tolerated microplastic pollution by improving the photosynthesis and antioxidant systems. Compared with conventional microplastic treatment, antioxidant systems may play a more critical role in generating the response against biodegradable microplastics. This is because a stronger relationship between maize growth and antioxidative biomarkers (GSH and CAT) was observed. In addition, the activities of GR and ascorbate peroxidase (APX) were enhanced in the presence of biodegradable microplastics. APX could utilize ascorbate as the electron donor to reduce H_2_O_2_ into H_2_O with the generation of monodehydroascorbate (MDHA), which can be further reduced to ASA. This reaction accompanies the transformation between GSH and glutathione disulfide (GSSG) [27]. Thus, the maize growth during exposure to biodegradable microplastics may be closely associated with the ascorbate‐glutathione (ASA‐GSH) cycle (Figure 1C). In contrast, against conventional microplastics, maize growth showed more associations with the process of photosynthesis. Photosynthesis is the foundation of plant growth, and improved photosynthetic performance can enhance the tolerance potential of plants to various stress factors [28, 29]. Although the results showed a connection between photosynthesis with alleviating conventional microplastic stress, the impacts of microplastics on plant photosynthesis remain debatable. Chai et al. reported a reduction in the net photosynthesis rates in mangroves during exposure to microplastics, such as small PE (13–150 μm), PP, and polyvinyl chloride (PVC), except for large PE microplastics (600 μm) where enhanced photosynthesis was observed [30]. Decreased net photosynthetic rates were also found in cucumber and soybean leaves during exposure to conventional microplastics [31, 32]. Colzi et al. observed that leaves of *Cucurbita pepo*, during exposure to PVC and PE treatments, showed a dose‐dependent reduction in the photosynthetic efficiency [33]. Thus, the photosynthetic parameters seem to be a dose–response and polymer‐dependent phenomenon of hormesis [34]. The mechanisms underlying the changes in photosynthesis are complex, which might relate to light energy absorption, dissipation, capture, and electron transfer, requiring further verification [35].

### Distinct transcriptomic alterations induced by biodegradable and conventional microplastics

**FIGURE 2:**
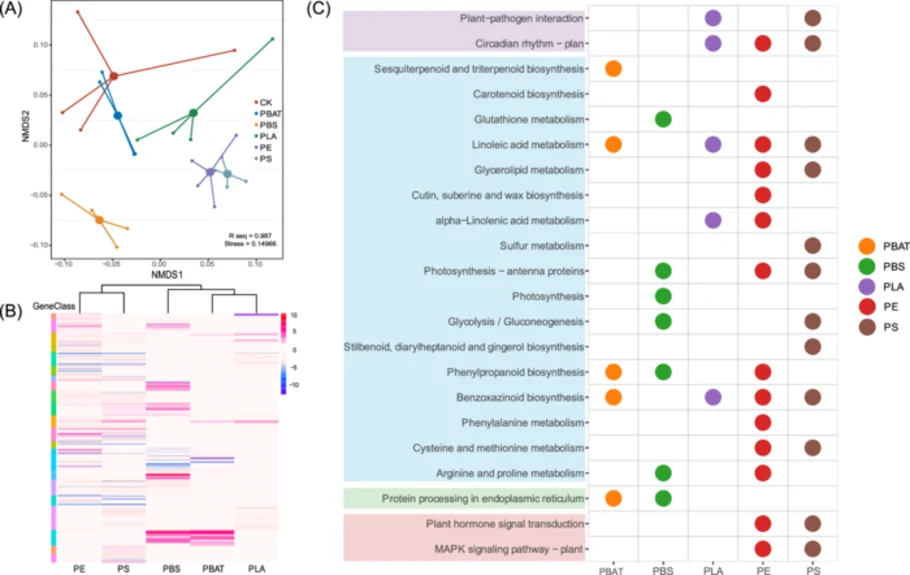
Responses of transcriptomic patterns of maize to microplastic. Nonmetric multidimensional scaling (NMDS) (based on Bray–Curtis distances) of all treatments (A). Heatmap visualizing log_2_‐transformed gene expression of differentially expressed genes (DEGs) (*p*‐value < 0.05 and |log_2_‐fold| change > 1) under different microplastic treatments (B). KEGG enrichment pathways of DEGs under different microplastic treatments (C).

To obtain the underlying insights into the responses of maize to the microplastics, we conducted a genomic‐transcriptome perspective on the leaf samples. We used nonmetric multi‐dimensional scaling (NMDS) analysis to visualize the clustering of gene expression in different treatments. The results showed that the conventional and biodegradable microplastic treatments were separated into distinct clusters (Figure 2A), indicating varied gene transcription after these treatments. We then identified the differentially expressed genes (DEGs) in the microplastic treatments in comparison with the control groups (Figure S5). More DEGs were observed in the conventional microplastic‐treated group. For example, PE treatment resulted in 1105 upregulated and 2320 downregulated genes, whereas PS treatment significantly upregulated 1239 and downregulated 2560 genes. For the biodegradable PBAT treatments, only 107 and 251 genes were significantly upregulated and downregulated, respectively. Clustering analysis based on log2‐transformed gene expression features also indicated that the maize transcriptomic responses to the biodegradable and conventional microplastics were separated into two distinct clusters (Figure 2B). These results potentially suggested that conventional microplastics might exhibit greater effects on the transcriptomic gene expression in maize leaves.

To understand the biological significance of DEGs, we carried out the functional enrichment analysis based on Kyoto Encyclopedia of Genes and Genomes (KEGG) annotations (Figures 2C, S6, and S7). Benzoxazinoid biosynthesis, phenylpropanoid biosynthesis, and photosynthesis‐antenna proteins were the most common response pathways identified when maize was exposed to microplastics. Additionally, the sesquiterpenoid and triterpenoid biosynthesis pathways uniquely responded to the PBAT treatment, whereas the glutathione metabolism pathway and carotenoid metabolism pathway uniquely responded to the PBS and PLA microplastics, respectively. These observations potentially indicated that the metabolism‐related pathways were significantly induced during exposure to the biodegradable microplastics.

Benzoxazinoids and phenylpropanoids are typical plant‐derived secondary metabolites involved in plant defense and have been demonstrated to be important in defense mechanisms against biotic and abiotic environmental stresses [36, 37, 38]. Essential genes in the phenylpropanoid biosynthesis pathway (*E1.11.1.7* and *TOGT1*) were also significantly altered, indicating regulation of the synthesis of both catalytic and antioxidant enzymes in maize, which are known to be critical for their defensive and metabolic mechanisms [39, 40]. The photosynthetic antenna systems are specialized for the absorption of photons and transfer of energy in the form of excited states or excitons to the photosynthetic reaction center, which performs one of the most fundamental tasks for plants [41]. The significant changes in the genes (*psbC* and *psbA*) are important for maize to improve photosynthesis under abiotic stresses [42]. These observations potentially suggested that genes in these pathways may be involved in generating resistive responses against biodegradable microplastics. When maize leaves were treated with conventional microplastics, plant hormone signal transduction, and MAPK signaling pathway‐plant pathways were unique to PE and PS treatments, wherein the MAPK signaling pathway gene (*ERF1*) and plant hormone signal transduction gene (*PYL*) were found to be highly expressed (Figure S8), suggesting a stress response of maize to conventional microplastics [43].

### Different metabolite profiling of maize in response to microplastic stress

**FIGURE 3:**
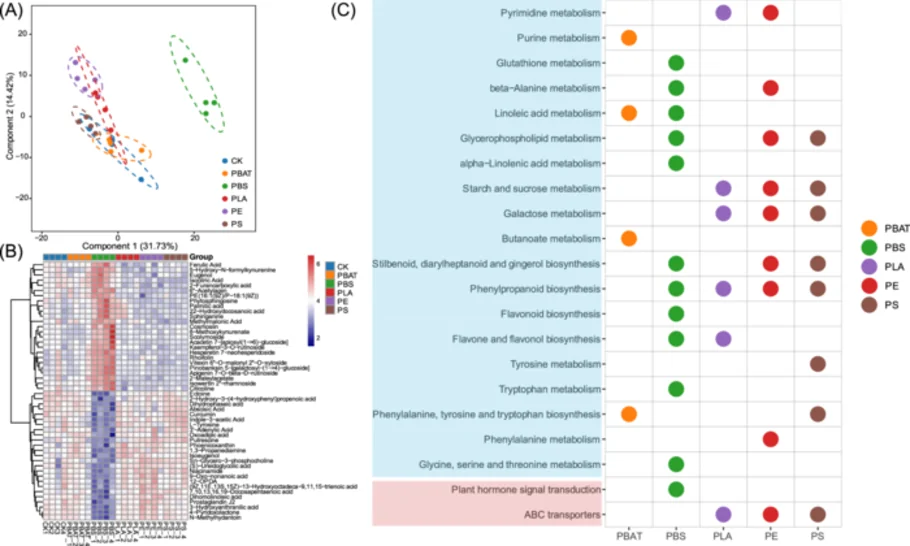
Responses of metabolome patterns of maize to microplastic. Sparse partial least‐squares discriminant analysis (sPLS‐DA) of maize metabolome. (A). Heatmap with the top 50 metabolites contributing to separation along with Components 1 and 2 (B). KEGG enrichment pathways of differentially expressed metabolites (DEMs) (*p*‐value < 0.05 and |log_2_‐fold| change > 1) under different microplastic treatments (C).

In parallel to the transcriptome profiling, we conducted the metabolic analysis of maize leaves using nontargeted metabolomics to identify the differences under microplastic treatments. A total of 933 metabolites were identified. The metabolomic profiles were first explored using sparse partial least‐squares discriminant analysis (sPLS‐DA) (Figure 3A). The score plot of sPLS‐DA analysis indicated that PBS microplastics and other treatments had a clear separation based on the global effects of changing metabolites. The metabolites after PBAT and PS microplastics treatments and the control were closely clustered, suggesting the limited effects of PBAT and PS on the metabolite profile of maize. We then selected the top 50 indicator metabolites that contributed to the variation along with components 1 and 2 of sPLS‐DA (Figure 3B and Figure S9), which dominantly belonged to flavonoids (18%), fatty acyls (16%) and carboxylic acids and derivatives (10%). Metabolites after PBS microplastic treatment showed significantly different patterns compared with those of control and other microplastic treatments. For example, flavonoid‐compounds including 6"‐acetylapiin, isowertin 2"‐rhamnoside, and pinobanksin 5‐[galactosyl‐(1‐>4)‐glucoside], were relatively higher in PBS‐treated maize compared with those in other groups. The DEseq. 2 R package was subsequently used to screen the differential metabolites (*p* adjust value < 0.05 and |log_2_‐fold change | > 1) in comparison with the control (Figure S10). PBS (141 upregulated and 81 downregulated) and PLA (38 upregulated and 22 downregulated) treatments had more differential metabolites than traditional PE (28 upregulated and 23 downregulated) and PS (23 upregulated and 10 downregulated) treatments, whereas, in a similar comparison, PBAT treatments identified fewer differential metabolites (13 upregulated and 12 downregulated).

The key metabolic pathways were further identified through the KEGG enrichment pathway analysis. As shown in Figure 3C, the phenylpropanoid biosynthesis pathway was enriched under different polymer treatments. Linoleic acid metabolism, flavone and flavonol biosynthesis, galactose metabolism (PLA and PE), and starch and sucrose metabolism pathways were significantly enriched in PBAT, PBS, PLA, PE, and PS treatment, respectively. These results were consistent with the differentially enriched metabolites found in the corresponding treatments (Figure S11). For example, flavonoids (e.g., baicalin and kaempferitrin) were significantly enriched in PBS microplastic‐treated maize, whereas sucrose 6‐phosphate was significantly enriched in PLA and PE‐treated maize. Plants have evolved complex mechanisms for the biosynthesis of secondary metabolites to cope with biotic stresses [44]. Flavonoids are the main class of secondary metabolites and can help plants protect against adverse environmental constraints [45]. Similar results were previously reported when plants were exposed to biotic or abiotic stress of Cd [46], salt [47], and PS microplastics [48]. Furthermore, we observed that maize leaves can respond to microplastic acclimation by enriching pathways related to amino acid, lipid, and carbohydrate metabolism to increase energy. Similar to other forms of life, amino acids are critical components for plant growth; besides their usage during protein biosynthesis, they also represent building blocks for several other biosynthesis pathways and play pivotal roles during signaling processes as well as during stress [49]. Under normal conditions, the oxidation of carbohydrates supplies respiration. However, under stress conditions, plant cells can modify their metabolism to utilize alternative respiratory substrates, for example, degradation of protein to free amino acids [50]. Primary metabolic processes, such as energy generation, carbohydrate metabolism, and amino acid metabolism, have been demonstrated to play vital roles in regulating plant defense responses and serve as a source of signaling molecules to directly or indirectly participate in plant defense responses [51, 52, 53]. Overall, the metabolic analysis indicated that the phenylpropanoid biosynthesis pathway was involved in the response of maize to the microplastic stress, which was consistent with the transcriptomic results.

### Molecular mechanisms of maize response to microplastic stress

**FIGURE 4:**
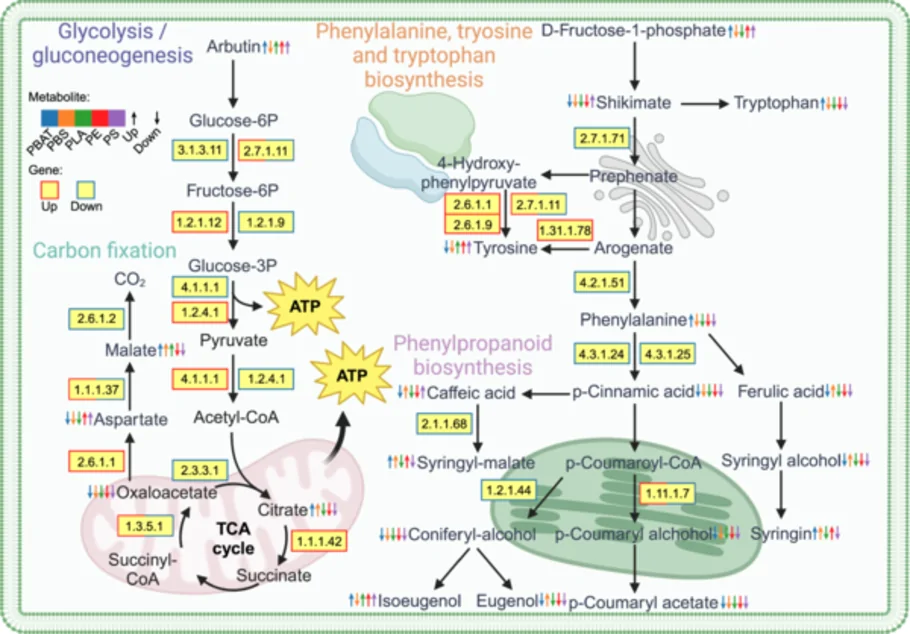
Expression levels of specific genes and metabolites in different treatments were mapped to the specific steps of glycolysis/gluconeogenesis, phenylalanine, tyrosine, and tryptophan biosynthesis, and its related pathways.

To further obtain insights into the mechanisms through which the microplastics impact the maize, we next performed an integrated analysis of transcriptomic and metabolomic results by selecting enriched pathways based on significantly differential genes and metabolites identified in maize in response to all treatments and constructed a co‐expression network of each pathway and its related branches (Figure S12). The results indicated that the phenylpropanoid biosynthesis; phenylalanine, tyrosine, and tryptophan biosynthesis; and glycolysis/gluconeogenesis pathways were identified as major players, highlighting the comprehensive regulation of these pathways in the response networks against all microplastics. In plants, aromatic amino acids (phenylalanine, tyrosine, and tryptophan) are precursors for a wide range of secondary metabolites, including phenylpropanoid, and these secondary metabolites have multiple functions, particularly protecting against various abiotic and biotic stresses [54]. Stress can induce energy deficit and hence activate pathways of carbohydrate metabolism and alternative glycolysis to maintain energy and carbon skeletons for vital metabolic processes [55]. Therefore, we mapped multiomics data obtained during exposure to microplastics to these two pathways (phenylalanine, tyrosine, and tryptophan biosynthesis and glycolysis/gluconeogenesis) to explore response modes (Figure 4).

Synthesis of aromatic amino acids (phenylalanine, tyrosine, and tryptophan) started with the alteration of d‐fructose‐1‐phosphate by the shikimate pathway. Five key enzymes involved in this pathway, including shikimate kinase (EC: 2.7.1.71), aspartate aminotransferase (EC: 2.6.1.1), tyrosine aminotransferase (EC: 2.6.1.5), histidinol‐phosphate aminotransferase (E.C:2.6.1.9), and arogenate dehydrogenase (EC: 1.3.1.78), were found to be altered in response to microplastics treatments. l‐phenylalanine and l‐tyrosine are both regulated from prephenate; prephenate synthesizes l‐phenylalanine either via phenylpyruvate or arogenate [56]. The contents of phenylalanine and tryptophan were downregulated, whereas tyrosine accumulated during microplastic treatment of maize, probably providing a self‐adjustment in response to abiotic stress. l‐phenylalanine can be converted into diverse aromatic compounds via the phenylpropanoid biosynthesis pathway [57]. The biosynthesis of phenylpropanoids entails a sequence of central enzyme‐regulated reactions, and the expression of genes for these enzymes was found to be significantly altered. For example, phenylalanine ammonia‐lyase (*PAL*, EC: 4.3.1.24) and 4‐coumarate‐CoA ligase (EC: 6.2.1.12) were downregulated, whereas peroxidase (EC: 1.11.1.7) genes were upregulated as well as downregulated. *PAL* catalyzes the transformation of phenylalanine into cinnamic acid, which serves as the precursor for all of the phenylpropanoids of secondary metabolism [58]. Cinnamate 4‐hydroxylase (C4L) and 4‐coumarate‐CoA ligase (4CL) then catalyze the conversion of cinnamic acid to p‐coumaroyl‐CoA, which is the precursor for many phenylpropanoid products [59]. The building blocks of lignin metabolites are derived from the phenylpropanoid pathway. Three major monolignols, p‐coumaryl, coniferyl, and sinapyl alcohol, form the lignin polymer through radical coupled polymerization [60]. These were also found to be significantly altered (content‐wise) during microplastic treatments. In summary, the phenylpropanoid pathway was found to be perturbated by microplastics, which potentially suggested that microplastics could impact the amount and composition of lignin, affect the physicochemical properties, and provoke weakened fibers and vessels that further negatively affect plant growth.

With regard to glycolysis/gluconeogenesis pathway, the content of arbutin involved in the glycolysis/gluconeogenesis metabolic pathway was significantly upregulated. Genes encoding 6‐phosphofructokinase (*PFK*, EC: 2.7.1.11), glyceraldehyde‐3‐phosphate dehydrogenase (*GAPDH*, EC:1.2.1.9), and pyruvate dehydrogenase E1 component (EC: 1.2.4.1) in glycolysis/gluconeogenesis pathway were significantly altered in response to microplastics. Glucose is catabolized to pyruvate by glycolytic enzymes, including *GAPDH*, and the pyruvate then enters the mitochondrion and is used to produce acetyl‐CoA. Acetyl‐CoA condenses with oxaloacetate to produce citrate to drive the citric acid (TCA cycle) [61]. Oxaloacetate can be generated from glucose‐derived pyruvate or glutamine catabolism through the TCA cycle, which was found to be altered in response to exposure to different microplastics. Oxaloacetate is reduced to malate by the carbon‐fixation pathway, and then malate is decarboxylated to pyruvate by malate dehydrogenase (*MDH*, EC: 1.1.1.37), which was also found to be downregulated in the current study. Our results show that microplastic exposure significantly altered the malate‐aspartate shuttle, a critical pathway for energy supply and ATP synthesis in maize. These changes suggest a reorientation of plant homeostasis to adapt to stress.

Collectively, phenylpropanoid biosynthesis; phenylalanine, tyrosine, and tryptophan biosynthesis; glycolysis/gluconeogenesis; and carbon fixation pathways were altered by microplastics. Simultaneously, we used the DIABLO framework to identify key metabolites and genes associated with a specific outcome to explore correlations across multiple omics datasets of representative pathways and maize antioxidant and photosynthetic characteristics [62]. As shown in Figure S13, we observed the strongest covariation between the genes and metabolites (*r* ^2^ = 0.91), followed by genes and maize physiological properties (*r* ^2^ = 0.87) and metabolites and physiological properties (*r* ^2^ = 0.87). Thus, DIABLO offers additional evidence for validating integrated omics data and maize physiological characteristics in response to microplastic stress. Additionally, we also noticed that the ABA concentration and plant hormone signal transduction were altered by microplastics, potentially suggesting an important role for ABA in microplastic stress resistance. Further studies with ABA treatments would be necessary for understanding the molecular mechanisms underlying maize responses to microplastic stress.

### Belowground factors mediated maize responses to microplastic stress

Due to their similar shapes to the natural soil particles, the microplastics would not induce substantial changes in soil physical properties. Therefore, we primarily focused on the changes in soil nutrients, DOM properties, and microbes. Microplastic addition significantly increased dissolved organic carbon (DOC) concentrations compared with the control, except PLA treatment (Table S4). For instance, the DOC concentrations in the other four microplastic treatments ranged from 67.7 to 139.1 mg/kg, which were statistically (*p* < 0.05) higher than the values in the control. The FI values in all treatments were lower than 1.4, suggesting that the terrestrial sources, such as plants and soil organic matter, dominantly contributed to the DOM [63]. Microplastic addition did not significantly change the three fluorescence indices (Figure S14). Five fluorescent components were identified by excitation‐emission matrix‐parallel factor analysis (EEM‐PARAFAC) model (Table S5), including two microbial‐origin humic‐like components (C1 and C2), one high‐molecular‐size humic‐like component (C3), and two terrestrially‐derived humic materials (C4 and C5). In comparison with the control, microplastic addition generally increases the relative abundances of C1, C2, and C3 components, and decreased C4 and C5 components. For instance, the mean value of the relative abundance of the C1 component in the control was 23.8%, while the values in the microplastic treatments ranged from 29.1% to 32.4%. The mean values of C4 and C5 components in the control were 15.3% and 25.9%, respectively, which were 1.5–8.5 fold higher than the values in microplastic treatments. The increase in microbial‐origin DOM fluorescent components under the five microplastic treatments may potentially suggest the alteration of microbial communities. Additionally, the concentrations of soil NH_4_^+^‐N and NO_3_^−^‐N were higher in most microplastic treatments, which was also probably due to the degradation of soil DOM.

**FIGURE 5:**
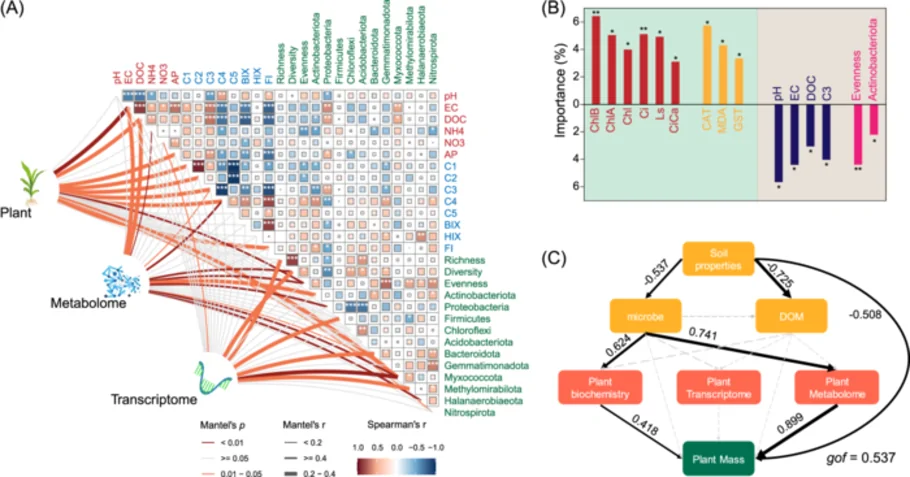
Correlations of the maize biochemical features, metabolome, and transcriptome with the soil variables, dissolved organic matter (DOM) fluorescent components, microbial alpha‐diversity, and the relative abundance of main phyla. Edge width corresponds to Mantel's *r* value, and the edge color denotes the statistical significance. Pairwise correlations of the variables are shown with a color gradient denoting Pearson's correlation coefficient (A). Contributions of the main above‐ground (i.e., maize photosynthetic performance and antioxidative responses) and below‐ground (i.e., soil properties, microbial alpha‐diversity, and the relative abundances of the main phyla) characteristics to maize mass based on random forest (RF) model (B). Partial least squares path model (PLS‐PM) shows the contribution of above‐ and below‐ground characteristics to plant mass (C). Numbers labeling the lines are indicative of the path coefficients. The solid lines indicate that the path coefficients are significant: *p* < 0.05.

After quality filtering, a total of 488,814 sequences was clustered into 5172 amplicon sequence variants (ASVs) across all soil samples. All treatments shared the most dominant phyla, including Actinobacteriota, Proteobacteria, Firmicutes, Chloroflexi, and Acidobacteriota, which accounted for 88.9%–91.5% of all taxa (Figure S15). Additionally, the abundances of the phylum Actinobacteriota were relatively lower in the microplastic treatments, and the phylum Proteobacteria was more abundant in the soils treated with microplastics. In comparison with the control, PBS microplastics significantly decreased the diversity and evenness of soil bacterial communities. A clear significant separation along the first principal component (18.79%) was observed between the control and the PBS treatment, while the separation along the second component (13.08%) was observed between the control and other microplastic treatments (Figure S15). To estimate the relationship among microbial community, soil variables, and DOM properties, the pairwise Spearman correlations were carried out (Figure 5). The DOC concentration was positively correlated with the C3 component and Proteobacteria and negatively correlated with BIX values. Additionally, the phylum Proteobacteria was negatively correlated with the alpha diversity indices. The results potentially suggested that microplastic addition can biasedly increase the relative abundance of Proteobacteria, and thus decrease the diversity and evenness of the soil bacterial communities. Furthermore, the strains belonged to Proteobacteria preferably utilize the labile DOM molecules and synthesize complex compounds [64], which may increase the humification degree of soil organic matter, confirming the negative correlation between Proteobacteria and BIX.

It is anticipated that the belowground characteristics would play important roles in driving the changes of plant performance. As shown in Figure 5, the maize biochemical features and metabolome were highly correlated with soil DOC concentration, DOM fluorescent components, the community evenness, and the relative abundance of the phylum Myxococcota. Maize transcriptome showed significant correlations with the richness and diversity of microbial communities and the relative abundances of the phyla Firmicutes, Bacteroidota, and Myxococcota. The results clearly indicated that the biochemical, metabolomic, and transcriptomic responses of the maize to microplastics would be closely related to the changes in the soil induced by microplastics. Subsequently, we performed RF modeling to identify the main predictors of the plant mass by estimating the importance of above‐ and below‐ground characteristics. We observed that the ChlB content, the antioxidant CAT enzyme, soil pH, and the microbial community evenness were important factors influencing the maize mass (Figure 5B). We further performed partial least‐squares‐path modeling (PLS‐PM) to quantify the contribution of above‐ and below‐ground characteristics to plant mass (Figure 5C). The overall fitness of the model was strong, with goodness‐of‐fit index (*GoF)* value of 0.537, suggesting that the important relationships were specified in this model. The plant mass was directly influenced by the soil chemical properties, maize biochemical responses, and metabolome. Significant effects of the soil microbial communities on maize biochemical responses and metabolome were also observed. Our results suggested that microplastics could affect plant performance directly by changing the soil environmental factors or indirectly by influencing the soil microbial communities.

Previous studies indicated that rhizosphere microorganisms could help plants to resist the abiotic stresses via mediate phytohormonal substances, for example, indole acetic acid, abscisic acid, and cytokinins [65, 66]. For instance, *Bacillus* could positively regulate the proline content, photosynthesis, and antioxidant enzyme activities of wheat tissue by producing indole acetic acid to alleviate salt stress [67]. In the current study, the plant hormone signal transduction and mitogen‐activated protein kinase signaling pathways were specifically regulated by microplastics, which was in agreement with the fact that plant‐associated microorganisms could enhance abiotic stress tolerance by regulating plant physiological activities. Additionally, microplastic‐induced changes in soil environmental variables could have direct impacts on plants, which augments previous reports on soil ecological effects of microplastics.

## 3 ENVIRONMENTAL IMPLICATION

**FIGURE 6:**
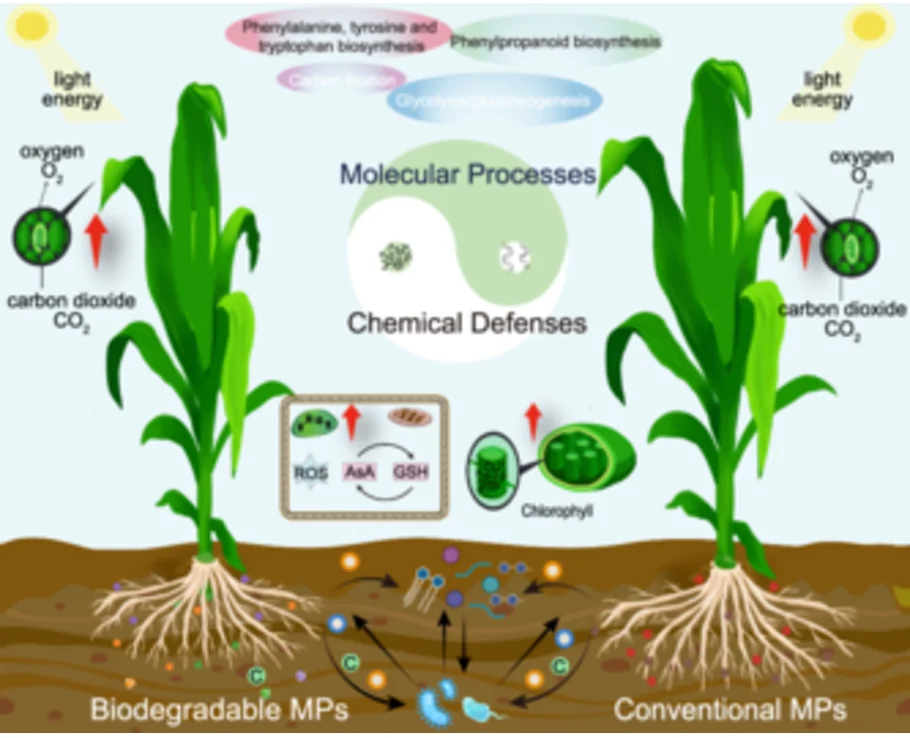
Schematic diagram of maize responses to biodegradable and conventional microplastics: Synergistic induction of molecular responses and chemical defenses, with growth influenced by alterations in soil environmental variables and microbial communities.

Since the use of biodegradable polymers will substantially increase in the future, these polymers will inevitably accumulate in the soil ecosystems (e.g., mulching, packaging, and littering). Thus, it is important to be aware of possible problems that may arise due to their use in agriculture or other fields. This study showed potential molecular mechanisms (at the metabolic and photosynthesis level) of the response of maize to five types of microplastics. Our results suggested that the biodegradable microplastics induced the functions of the ASA‐GSH cycle. Furthermore, enhanced photosynthetic performance in maize exposed to conventional microplastics plays a vital role. Multiomics analysis revealed that secondary metabolite biosynthesis, photosynthesis, energy metabolism, and carbohydrate metabolism were regulated in maize during the microplastic stress. Although the growth characteristics of maize showed limited changes under certain microplastic treatments, oxidative damages and molecular disturbances of maize leaves were evident. Additionally, our results indicated that the changes in belowground characteristics induced by microplastics may tiger the impacts on plant traits and function. Our findings comprehensively identified the molecular processes and chemical defense systems that act synergistically to adapt maize to microplastic stress (Figure 6).

What we should notice is that our study has some limitations. First, the exposure period was short, and the maize was not seeded. Second, it is not clear at this stage whether the observed metabolic changes influence the maize status (in terms of yield and quality). Field experiments were not conducted to evaluate the full life cycle of plants in the presence of microplastics. Additionally, the plant genotype may also act as a main driver of the molecular responses to microplastics. How genomic basis mediates microplastic adaptation of maize, particularly the relationship between host genetic variation and observed phenotypic differences among genotypes, remains elusive. Finally, we cannot simplistically frame biodegradable plastics as a new contaminant and study their ecotoxicological effects, as any carbon‐rich material can directly or indirectly influence the plant. However, we can underscore the potential perturbations induced by microplastics in plants, which should be comprehensively investigated, and the consequent effects should not be overlooked. Therefore, further studies that can potentially solve the above problems with biodegradable polymers are necessary.

## 4 METHODS

### Preparation of soil, microplastics, and plant materials

The surface soil (0–20 cm) was collected from an uncultured farmland without film mulching or known plastic pollution in the Shangzhuang Experimental Station at China Agricultural University in Beijing, China. We air‐dried the soil at room temperature (25°C) for 3 weeks and sieved it through a 2‐mm mesh to remove stones and plant residues. The soil physicochemical properties can be found in the (Table S1). Five typical and extensively applied plastics were selected in this study, including two types of conventional plastics PE and PS, and three types of biodegradable plastics PBAT, PBS, and PLA [15, 68]. PE and PS particles were purchased from Aladdin, whereas PBAT, PBS, and PLA particles were purchased from XingWang Plastic. These particles were sieved to the size range of 150–180 μm (80–100 mesh), cleaned with methanol, dried at 40°C, and stored at 4°C for further use. Fourier transform infrared spectroscopy and scanning electron microscopy (SEM) were used to characterize the particles (Figures S1 and S2). SEM showed that these microplastics were irregularly spherical particles and fragments. Maize seeds (Xianyu 335) were sterilized with 5% sodium hypochlorite solution for 5 min and rinsed with sterile deionized water before sowing.

### Pot experiment

The experimental design included five microplastic types (PBAT, PBS, PLA, PE, and PS), and a concentration of 1% (w/w) was used, which can be considered environmentally relevant for the soil with great human activities, as per previous studies [69]. Each ceramic pot contained 2.0 kg of soil mixed with or without 20 g of microplastic particles. Four seeds of maize were planted in each pot, and the plants were thinned to one per pot after the emergence of the third leaf. Each treatment included four replicates. The experiment was carried out in the greenhouse at China Agricultural University under natural conditions. The temperature in the greenhouse ranged from 19.5°C to 27.8°C, and the humidity was approximately 12%. During the experiment, the pots were weighted and watered with deionized water 2–3 times a week to maintain the field water holding capacity of approximately 20%.

### Plant measurement

At the end of the experiment (60 days), we recorded the plant height, stem width, and fresh biomass. Plant height was measured from the soil surface to the uppermost leaf being extended for a maximum height, and the stem thickness was measured at the soil surface as the widest diameter of the stalk [70]. Pn, Ls, Gs, Ci, and Tr of the most fully expanded fresh leaves were measured between 9:00 and 11:00 am on the last day, using a Li‐6400 portable photosynthetic system (LI‐COR) as described previously [71]. LWUEint and LWUEins were calculated following the methods described in the SI (Text S1). After these measurements, leaves were harvested and instantly placed into 50 mL conical centrifuge tubes and stored at −80°C.

### Biochemical assay

Leaf samples were homogenized in 10 mmol/L phosphate buffer (pH 7.4) at a 1:10 (mass: volume) ratio for 1 min using a PRO200 homogenizer at 10,000 rpm at 4°C. The homogenate was subsequently centrifuged at 3500 × *g* for 10 min at 4°C, and the supernatant was used for the biochemical assays. The protein concentration was measured using a Coomassie Brilliant Blue protein assay kit (Nanjing Jiancheng Bioengineering Institute) following the manufacturer's instructions. The activities of antioxidant enzymes, including CAT, peroxidase (POD), polyphenol oxidase (PPO), GR, APX, and glutathione S‐transferase (GST) were measured using specific assay kits (Nanjing Jiancheng Bioengineering Institute, Nanjing, China) employing UV–Vis spectrophotometer (Shimadzu UV‐1900i, Japan) or microplate reader (Epoch‐SN, BioTek) according to the manufacturer's instructions. Biomarkers of lipid peroxidation (MDA) and nonenzymatic antioxidants (ASA, GSH, and ABA) were also measured using assay kits (Nanjing Jiancheng Bioengineering Institute). The colorimetric analysis of chlorophyll a and b was performed at 645 and 663 nm, respectively, with a UV–Vis spectrophotometer (Shimadzu UV‐1900i, Japan).

### Transcriptome analysis

The total RNA of maize leaf was extracted using the RNAQUEOUS kit (Ambion‐1912) following the manufacturer's introduction. Thermo Scientific's NanoDrop 2000 spectrophotometer was used to calculate the RNA amount, whereas an agarose gel electrophoresis stained with ethidium bromide was performed to assess the integrity of the samples. The concentration and purity of the extracted total RNA were further analyzed on an Agilent 2100 bioanalyzer. The samples with high quality were selected for transcriptome sequencing. Transcriptomic analysis was performed by Majorbio Bio‐Pharm Technology Co., Ltd (Shanghai, China) using the Illumina HiSeq. 6000 platform (Illumina Inc.) with 150 bp paired‐end reads. Trimmmatic software was used to preprocess the quality of the original data (to remove the adapter, low‐quality reads, and low‐quality bases). High‐quality reads were mapped to the reference genome sequence using Tophat2 tools [72]. The mapped reads were assembled using the StringTie assembly tool (http://ccb.jhu.edu/software/stringtie/) [73]. The assembled results were annotated based on Gene Ontology (GO) and KEGG databases. Gene expression levels were quantified as the values of transcripts per million reads calculated using RESM software [74]. DESeq. 2R package (1.40.2) was used to identify the DEGs using a |log_2_‐fold change| of >1 and a *p* adjust value of <0.05. The p values were adjusted using Benjamini and Hochberg's approach for controlling the false discovery rate [75].

### Metabolomic analysis

Maize leaves were ground in 75% aqueous methanol using a high‐throughput tissue grinder. Leaves extract was subsequently ultrasonicated for 30 min in an ice bath and stored at −20°C for 30 min. Sample extracts were centrifuged at 13,000 rpm for 15 min at 4°C, and the supernatant was transferred to 1 mL vials. LC‐MS was performed on a Thermo UHPLC system equipped with a binary solvent delivery manager and a sample manager, coupled with a Thermo Q Exactive Mass Spectrometer equipped with an electrospray interface. An Acquity BEH C18 column (100 mm × 2.1 mm i.d., 1.7 μm; Waters) was used. The injection volume was 10 μL, and the flow rate was 0.4 mL/min with column temperature at 40°C. Table S2 describes the gradient elution program. The mass spectrometric data were collected using a Thermo UHPLC‐Q Exactive Mass Spectrometer equipped with an electrospray ionization source operating in both positive and negative ion mode with a mass range of 70–1050 m/z. Text S2 describes the mass spectrometric analysis and annotation of metabolites.

### DOM spectroscopic characterization and amplicon sequencing of rhizosphere soil

The maize roots were removed from the soil. The excess soil was manually shaken from the roots, leaving approximately 2 mm of soil still attached to the roots. We then vigorously stirred the roots with sterile forceps to separate the 2 mm of soil from the root surfaces, termed as the rhizosphere soil. A number of chemical variables in each rhizosphere soil were determined, including pH, electrical conductivity (EC), DOC, ammonium (NH_4_^+^‐N), nitrate (NO_3_^−^‐N), and available phosphorus (AP). All of the details were given in SI. The DOM of the rhizosphere soil was extracted using ultrapure water at a soil/water ratio of 1:10 (w/v). An Aqualog Analyzer (HORIBA Scientific) was used for the EEM analysis. The excitation wavelength was set from 250 to 550 nm at 5 nm increments, and the emission spectra ranged from 280 to 600 nm at 2 nm intervals. The optical indices, including humification index (HIX), biological index (BIX), and fluorescence index (FI), were calculated. PARAFAC was further performed to quantify the fluorescent composition. Details about the calculation of fluorescent indices and PARAFAC were given in SI.

The DNA of rhizosphere soil was extracted using the MoBio PowerSoil DNA isolation kit (Qiagen, Shanghai, China). The V3–V4 region of the 16S rRNA gene was amplified by using the primer pair 338F (ACTCCTACGGGAGGCAGCA) and 806R (GGACTACHVGGGTWTCTAAT). The amplified products were sequenced in a 2 × 300‐bp paired‐end format using the Illumina MiSeq platform at Majorbio BioPharm Technology Co. Ltd. The standard QIIME2 (Quantitative Insights Into Microbial Ecology, version 2024.2.0) pipeline was used to process the raw 16S rRNA gene sequences. The DADA2 plugin was used to trim and denoise the raw reads, and clustered them into ASVs. The taxonomic information of each representative ASV was assigned using the SILVA reference database (Version 138). Details about the amplification process and bioinformation analysis were given in SI.

### Statistical analysis

All statistical and graphical analysis was performed in the R environment (v 4.2.2; R Core Team, 2023), and graphs were constructed using the “ggplot2” package unless otherwise stated [76]. PCA (R factoextra package), NMDS (R vegan package), and Principal Coordinate Analysis (PCoA, R vegan package) were used to perform dimension‐reduced analysis. Nonparametric multivariate analysis of variance (Adonis) was used to test the significance in different treatments. sPLS‐DA was conducted using the R package mixOmics, and the function of DIABLO in this package was used to generate integrated omics data and maize physiological characteristics. Pathway interaction networks were built using Cytoscape v3.9.0. To link the belowground variables (soil chemical properties, DOM parameters, and microbial community) and plant characteristics, a partial Mantel test with 999 permutations was carried out. To evaluate the relative importance of belowground variables and plant physiological characteristics contributing to plant mass, random forest analysis was conducted with the “randomForest” package in R (ntree = 1000). To further discern the direct and indirect effects of the belowground variables on plant mass, a PLS‐PM was conducted with the “plspm” package in R. The overall prediction performance of this mode was validated using the *GoF*, with a higher value indicating better prediction performance. Analysis was deemed significant if *p*‐value was <0.05. The statistical significance was analyzed using a one‐way analysis of variance followed by the least significant difference test. The results are presented as the means ± standard deviations.

## AUTHOR CONTRIBUTIONS

**Yuanze Sun**: Writing—original draft; formal analysis; validation; writing—review and editing; visualization. **Jingxi Zang**: Validation; writing—review and editing. **Siyuan Xie**: Validation; visualization; formal analysis. **Mochen Wu**: Validation; writing—review and editing; formal analysis. **Jianguo Tao**: Validation; formal analysis. **Tanveer M. Adyel**: Writing—review and editing; funding acquisition. **Xinyu Du**: Writing—review and editing; funding acquisition. **Si Li**: Writing—review and editing. **Jie Wang**: Conceptualization; funding acquisition; writing— review and editing; project administration; supervision. All authors have read the final manuscript and approved it for publication.

## CONFLICT OF INTEREST STATEMENT

The authors declare no conflicts of interest.

## ETHICS STATEMENT

No animals or humans were involved in this study.

## Supporting information

The online version contains supplementary figures and tables available.

**Figure S1.** The fourier transform infrared spectroscopy (FTIR) results of the five microplastics particles.
**Figure S2.** The scanning electron microscopy (SEM) photos of the five microplastic particles.
**Figure S3.** Responses of antioxidant defense system and photosynthetic pigment of maize to microplastic.
**Figure S4.** Mantel test analyzed the relationship between the phenotypic changes of maize and antioxidant and photosynthetic index in (A) all treatments, (B) biodegradable microplastic treatments, (C) conventional microplastic treatment.
**Figure S5.** Volcano plot of differentially expressed genes (DEGs) in maize between the control and microplastic treatments.
**Figure S6.** Kyoto Encyclopedia of Genes and Genomes (KEGG) enrichment analysis of DEGs in different treatments.
**Figure S7.** Venn diagram of DEGs in different microplastics treatments (A). KEGG pathway analysis of unique DEGs in different microplastics treatments (B).
**Figure S8.** *PTL*, *ERF1*, *HSP20*, and *GAPDH* gene expression in different treatments and the control.
**Figure S9.** The metabolites contributing to separation along with Component 1 and 2 based on sparse partial least‐squares discriminant analysis (sPLS‐DA) (A), and the functional pathways of the biomarker metabolites (B).
**Figure S10.** Volcano plot of differentially expressed metabolites (DEMs) in maize between the control and microplastic treatments.
**Figure S11.** KEGG enrichment analysis of DEMs in different treatments.
**Figure S12.** Transcription−metabolism regulation integration network of enriched pathways in different treatments.
**Figure S13.** DIABLO analysis demonstrated the relationship between antioxidant and photosynthetic parameters, representative pathway genes, and metabolites.
**Figure S14.** The DOM properties in soil under different treatments.
**Figure S15.** The bacterial communities in soil under different treatments.

**Table S1.** The physicochemical properties of soil used in this study.
**Table S2.** Elution gradient of mobile phase that used for UPLC‐MS/MS analyses.
**Table S3.** Photosynthetic parameters of maize in different treatments.
**Table S4.** Soil properties under different treatments.
**Table S5.** Description of five component parallel factor analysis (PARAFAC) model.

## Data Availability

The raw sequence data of maize transcriptome and soil rRNA gene were deposited in the Genome Sequence Archive of China National Genomics Data Center under accession number PRJCA032010 and PRJCA029794, respectively. The data and scripts used are saved in GitHub https://github.com/watertimes/maize_and_microplastics. Supplementary materials (figures, tables, graphical abstract, slides, videos, Chinese translated version, and update materials) may be found in the online DOI or for iMetaOmics http://www.imeta.science/imetaomics/.

## References

[1] Andrady, Anthony L . 2011. “Microplastics in the Marine Environment.” Marine Pollution Bulletin 62: 1596–1605. 10.1016/j.marpolbul.2011.05.030 21742351

[2] Li, Jingyi , Huihui Liu , and J. Paul Chen . 2018. “Microplastics in Freshwater Systems: A Review on Occurrence, Environmental Effects, and Methods for Microplastics Detection.” Water Research 137: 362–374. 10.1016/j.watres.2017.12.056 29580559

[3] Zhang, Yulan , Shichang Kang , Steve Allen , Deonie Allen , Tanguang Gao , and Mika Sillanpää . 2020. “Atmospheric Microplastics: A Review on Current Status and Perspectives.” Earth‐Science Reviews 203: 103118. 10.1016/j.earscirev.2020.103118

[4] de Souza Machado, Anderson Abel , Chung Wai Lau , Jennifer Till , Werner Kloas , Anika Lehmann , Roland Becker , and Matthias C. Rillig . 2018. “Impacts of Microplastics on the Soil Biophysical Environment.” Environmental Science & Technology 52: 9656–9665. 10.1021/acs.est.8b02212 PMC6128618 30053368

[5] Ambrosini, Roberto , Roberto Sergio Azzoni , Francesca Pittino , Guglielmina Diolaiuti , Andrea Franzetti , and Marco Parolini . 2019. “First Evidence of Microplastic Contamination in the Supraglacial Debris of an Alpine Glacier.” Environmental Pollution 253: 297–301. 10.1016/j.envpol.2019.07.005 31323612

[6] González‐Pleiter, Miguel , Carlos Edo , Ángeles Aguilera , Daniel Viúdez‐Moreiras , Gerardo Pulido‐Reyes , Elena González‐Toril , Susana Osuna , et al. 2021. “Occurrence and Transport of Microplastics Sampled Within and Above the Planetary Boundary Layer.” Science of the Total Environment 761: 143213. 10.1016/j.scitotenv.2020.143213 33162145

[7] Huang, Yi , Qin Liu , Weiqian Jia , Changrong Yan , and Jie Wang . 2020. “Agricultural Plastic Mulching As a Source of Microplastics in the Terrestrial Environment.” Environmental Pollution 260: 114096. 10.1016/j.envpol.2020.114096 32041035

[8] Nizzetto, Luca , Gianbattista Bussi , Martyn N. Futter , Dan Butterfield , and Paul G. Whitehead . 2016. “A Theoretical Assessment of Microplastic Transport in River Catchments and their Retention by Soils and River Sediments.” Environmental Science: Processes & Impacts 18: 1050–1059. 10.1039/C6EM00206D 27255969

[9] Evangeliou, N. , H. Grythe , Z. Klimont , C. Heyes , S. Eckhardt , S. Lopez‐Aparicio , and A. Stohl . 2020. “Atmospheric Transport is a Major Pathway of Microplastics to Remote Regions.” Nature Communications 11: 3381. 10.1038/s41467-020-17201-9 PMC7360784 32665541

[10] de Souza Machado, Anderson Abel , Chung W. Lau , Werner Kloas , Joana Bergmann , Julien B. Bachelier , Erik Faltin , Roland Becker , Anna S. Görlich , and Matthias C. Rillig . 2019. “Microplastics Can Change Soil Properties and Affect Plant Performance.” Environmental Science & Technology 53: 6044–6052. 10.1021/acs.est.9b01339 31021077

[11] Huang, Yi , Yanran Zhao , Jie Wang , Mengjun Zhang , Weiqian Jia , and Xiao Qin . 2019. “LDPE Microplastic Films Alter Microbial Community Composition and Enzymatic Activities in Soil.” Environmental Pollution 254: 112983. 10.1016/j.envpol.2019.112983 31394342

[12] Sun, Yuanze , Chongxue Duan , Na Cao , Changfeng Ding , Yi Huang , and Jie Wang . 2022. “Biodegradable and Conventional Microplastics Exhibit Distinct Microbiome, Functionality, and Metabolome Changes in Soil.” Journal of Hazardous Materials 424: 127282. 10.1016/j.jhazmat.2021.127282 34607029

[13] Mamun, Abdullah Al , Tofan Agung Eka Prasetya , Indiah Ratna Dewi , and Monsur Ahmad . 2023. “Microplastics in Human Food Chains: Food Becoming a Threat to Health Safety.” Science of the Total Environment 858: 159834. 10.1016/j.scitotenv.2022.159834 36461575

[14] Mukherjee, Chandrapaul , Dissa Varghese , J. S. Krishna , T. Boominathan , R. Rakeshkumar , S. Dineshkumar , C. V. S. Brahmananda Rao , and Akella Sivaramakrishna . 2023. “Recent Advances in Biodegradable Polymers – Properties, Applications and Future Prospects.” European Polymer Journal 192: 112068. 10.1016/j.eurpolymj.2023.112068

[15] “European bioplastics: Bioplastics market data https://www.european-bioplastics.org/market/.”

[16] Liao, Jin , and Qiqing Chen . 2021. “Biodegradable Plastics in the Air and Soil Environment: Low Degradation Rate and High Microplastics Formation.” Journal of Hazardous Materials 418: 126329. 10.1016/j.jhazmat.2021.126329 34118549

[17] Wei, Xin‐Feng , Martin Bohlén , Catrin Lindblad , Mikael Hedenqvist , and Aron Hakonen . 2021. “Microplastics Generated from a Biodegradable Plastic in Freshwater and Seawater.” Water Research 198: 117123. 10.1016/j.watres.2021.117123 33865028

[18] Yang, Chong , and Xuhua Gao . 2022. “Impact of Microplastics from Polyethylene and Biodegradable Mulch Films on Rice (Oryza sativa L.).” Science of the Total Environment 828: 154579. 10.1016/j.scitotenv.2022.154579 35302020

[19] Brown, Robert W. , David R. Chadwick , Huadong Zang , Martine Graf , Xuejun Liu , Kai Wang , Lucy M. Greenfield , and Davey L. Jones . 2023. “Bioplastic (PHBV) Addition to Soil Alters Microbial Community Structure and Negatively Affects Plant‐Microbial Metabolic Functioning in Maize.” Journal of Hazardous Materials 441: 129959. 10.1016/j.jhazmat.2022.129959

[20] Wang, Quanlong , Xueying Feng , Yingying Liu , Wenguang Li , Wenzhi Cui , Yuhuan Sun , Shuwu Zhang , Fayuan Wang , and Baoshan Xing . 2023. “Response of Peanut Plant and Soil N‐Fixing Bacterial Communities to Conventional and Biodegradable Microplastics.” Journal of Hazardous Materials 459: 132142. 10.1016/j.jhazmat.2023.132142 37515992

[21] Wang, Jia Lin , Kai Lou Liu , Xue Qiang Zhao , Hao Qing Zhang , Dong Li , Jiao Jiao Li , and Ren Fang Shen . 2021. “Balanced Fertilization over Four Decades Has Sustained Soil Microbial Communities and Improved Soil Fertility and Rice Productivity in Red Paddy Soil.” Science of the Total Environment 793: 148664. 10.1016/j.scitotenv.2021.148664 34328991

[22] Qu, Qian , Zhenyan Zhang , W. J. G. M. Peijnenburg , Wanyue Liu , Tao Lu , Baolan Hu , Jianmeng Chen , et al. 2020. “Rhizosphere Microbiome Assembly and Its Impact on Plant Growth.” Journal of Agricultural and Food Chemistry 68: 5024–5038. 10.1021/acs.jafc.0c00073 32255613

[23] Chen, Qing‐Lin , Jing Ding , Yong‐Guan Zhu , Ji‐Zheng He , and Hang‐Wei Hu . 2020. “Soil Bacterial Taxonomic Diversity is Critical to Maintaining the Plant Productivity.” Environment International 140: 105766. 10.1016/j.envint.2020.105766 32371308

[24] Ngoune Tandzi, Liliane , and Charles Shelton Mutengwa . 2020. “Estimation of Maize (Zea mays L.) Yield Per Harvest Area: Appropriate Methods.” Agronomy 10: 29. 10.3390/agronomy10010029

[25] Andrady, Anthony L. , and Mike A. Neal . 2009. “Applications and Societal Benefits of Plastics.” Philosophical Transactions of the Royal Society B: Biological Sciences 364: 1977–1984. 10.1098/rstb.2008.0304 PMC2873019 19528050

[26] Chinthapalli, Raj , Pia Skoczinski , Michael Carus , Wolfgang Baltus , Doris de Guzman , Harald Käb , Achim Raschka , and Jan Ravenstijn . 2019. “Biobased Building Blocks and Polymers—Global Capacities, Production and Trends, 2018–2023.” Industrial Biotechnology 15: 237–241. 10.1089/ind.2019.29179.rch

[27] Foyer, Christine H. , and Graham Noctor . 2011. “Ascorbate and Glutathione: The Heart of the Redox Hub.” Plant Physiology 155: 2–18. 10.1104/pp.110.167569 PMC3075780 21205630

[28] Tankari, Moussa , Chao Wang , Haiyang Ma , Xiangnan Li , Li Li , Rajesh Kumar Soothar , Ningbo Cui , et al. 2021. “Drought Priming Improved Water Status, Photosynthesis and Water Productivity of Cowpea During Post‐Anthesis Drought Stress.” Agricultural Water Management 245: 106565. 10.1016/j.agwat.2020.106565

[29] Yang, Y. , L. Q. Zhang , X. Huang , Y. Y. Zhou , Q. M. Quan , Y. X. Li , and X. H. Zhu . 2020. “Response of Photosynthesis to Different Concentrations of Heavy Metals in *Davidia involucrata* .” PloS One 15: e0228563. 10.1371/journal.pone.0228563 PMC7075629 32176700

[30] Chai, Minwei , Ruili Li , Bing Li , Hailun Wu , and Lingyun Yu . 2023. “Responses of Mangrove (*Kandelia obovata*) Growth, Photosynthesis, and Rhizosphere Soil Properties to Microplastic Pollution.” Marine Pollution Bulletin 189: 114827. 10.1016/j.marpolbul.2023.114827 36931158

[31] Zhuang, Haoran , Xiaoyi Liu , Han Ma , Ruijing Li , Bo Liu , Ziyu Lin , and Zhenxia Li . 2023. “Growth and Physiological–Biochemical Characteristics of Cucumber (Cucumis sativus L.) in the Presence of Different Microplastics.” Arabian Journal of Geosciences 16: 194. 10.1007/s12517-023-11273-9

[32] Li, Haibin , Fupeng Song , Xiliang Song , Kongming Zhu , Qun Lin , Jinliang Zhang , and Guoqiang Ning . 2023. “Single and Composite Damage Mechanisms of Soil Polyethylene/Polyvinyl Chloride Microplastics to the Photosynthetic Performance of Soybean (Glycine max [L.] Merr.).” Frontiers in Plant Science 13: 1100291. 10.3389/fpls.2022.1100291 PMC9889878 36743543

[33] Colzi, Ilaria , Luciana Renna , Elisabetta Bianchi , Maria Beatrice Castellani , Andrea Coppi , Sara Pignattelli , Stefano Loppi , and Cristina Gonnelli . 2022. “Impact of Microplastics on Growth, Photosynthesis and Essential Elements in Cucurbita pepo L.” Journal of Hazardous Materials 423: 127238. 10.1016/j.jhazmat.2021.127238 34844356

[34] Agathokleous, Evgenios , ZhaoZhong Feng , Ivo Iavicoli , and Edward J. Calabrese . 2019. “The Two Faces of Nanomaterials: A Quantification of Hormesis in Algae and Plants.” Environment International 131: 105044. 10.1016/j.envint.2019.105044 31362152

[35] Teng, Linhong , Yihao Zhu , Haibin Li , Xiliang Song , and Lianhui Shi . 2022. “The Phytotoxicity of Microplastics to the Photosynthetic Performance and Transcriptome Proﬁling of *Nicotiana Tabacum* Seedlings.” Ecotoxicology and Environmental Safety 231: 113155. 10.1016/j.ecoenv.2021.113155 35007831

[36] Su, Xiaoyu , Longfei Xin , Zhuo Li , Huifang Zheng , Jun Mao , and Qinghua Yang . 2018. “Physiology and Transcriptome Analyses Reveal a Protective Effect of the Radical Scavenger Melatonin in Aging Maize Seeds.” Free Radical Research 52: 1094–1109. 10.1080/10715762.2018.1472378 29722273

[37] Niemeyer, Hermann M . 2009. “Hydroxamic Acids Derived from 2‐hydroxy‐2 H‐1, 4‐benzoxazin‐3 (4 H)‐One: Key Defense Chemicals of Cereals.” Journal of Agricultural and Food Chemistry 57: 1677–1696. 10.1021/jf8034034 19199602

[38] Zhou, Shaoqun , Annett Richter , and Georg Jander . 2018. “Beyond Defense: Multiple Functions of Benzoxazinoids in Maize Metabolism.” Plant and Cell Physiology 59: 1528–1537. 10.1093/pcp/pcy064 29584935

[39] Zhao, Chen‐Yu , Shaoguo Ru , Pengfei Cui , Xin Qi , Mayur B. Kurade , Swapnil M. Patil , Byong‐Hun Jeon , and Jiu‐Qiang Xiong . 2021. “Multiple Metabolic Pathways of Enrofloxacin By *Lolium Perenne* L.: Ecotoxicity, Biodegradation, and Key Driven Genes.” Water Research 202: 117413. 10.1016/j.watres.2021.117413 34271457

[40] Sam, O. , M. Núñez , M. C. Ruiz‐Sánchez , J. Dell'Amico , V. Falcón , M. C. De la Rosa , and J. Seoane . 2001. “Effect of a Brassinosteroid Analogue and High Temperature Stress on Leaf Ultrastructure of Lycopersicon Esculentum.” Biologia Plantarum 44: 213–218. 10.1023/A:1010291124178

[41] Dorfman, Konstantin E. , Dmitri V. Voronine , Shaul Mukamel , and Marlan O. Scully . 2013. “Photosynthetic Reaction Center as a Quantum Heat Engine.” Proceedings of the National Academy of Sciences 110: 2746–2751. 10.1073/pnas.1212666110 PMC3581952 23365138

[42] Li, Hui , Xuewu He , Yuanfen Gao , Wenjuan Liu , Jun Song , and Junjie Zhang . 2023. “Integrative Analysis of Transcriptome, Proteome, and Phosphoproteome Reveals Potential Roles of Photosynthesis Antenna Proteins in Response to Brassinosteroids Signaling in Maize.” Plants 12: 1290. 10.3390/plants12061290 PMC10058427 36986978

[43] Danquah, Agyemang , Axel de Zelicourt , Jean Colcombet , and Heribert Hirt . 2014. “The Role of ABA and MAPK Signaling Pathways in Plant Abiotic Stress Responses.” Biotechnology Advances 32: 40–52. 10.1016/j.biotechadv.2013.09.006 24091291

[44] Patra, Barunava , Craig Schluttenhofer , Yongmei Wu , Sitakanta Pattanaik , and Ling Yuan . 2013. “Transcriptional Regulation of Secondary Metabolite Biosynthesis in Plants.” Biochimica et Biophysica Acta (BBA)‐Gene Regulatory Mechanisms 1829: 1236–1247. 10.1016/j.bbagrm.2013.09.006 24113224

[45] Kumar, Vinay , Rubal Upsana Suman , and Sudesh Kumar Yadav . 2018. “Flavonoid Secondary Metabolite: Biosynthesis and Role in Growth and Development in Plants.” In Recent Trends and Techniques in Plant Metabolic Engineering, edited by S. Yadav , V. Kumar , and S. Singh , 19–45. 10.1007/978-981-13-2251-8_2

[46] Wei, Zhao , Chen Zhongbing , Yang Xiuqin , Sheng Luying , Mao Huan , and Zhu Sixi . 2023. “Integrated Transcriptomics and Metabolomics Reveal Key Metabolic Pathway Responses in Pistia stratiotes under Cd Stress.” Journal of Hazardous Materials 452: 131214. 10.1016/j.jhazmat.2023.131214 36989786

[47] Wang, Wei , Jinfeng Cao , Sufang Huang , Zhi Wang , Weiwei Wang , Jingwei Zou , Fengzhi Wang , Meng Luo , and Jingjian Zhang . 2023. “Integrated Transcriptomics and Metabolomics Analyses Provide Insights Into Salt‐Stress Response in Germination and Seedling Stage of Wheat (Triticum aestivum L.).” Current Plant Biology 33: 100274. 10.1016/j.cpb.2023.100274

[48] Sun, Shan , Xiangang Hu , Weilu Kang , and Mingqi Yao . 2023. “Combined Effects of Microplastics and Warming Enhance Algal Carbon and Nitrogen Storage.” Water Research 233: 119815. 10.1016/j.watres.2023.119815 36881974

[49] Hildebrandt, Tatjana M. , Adriano Nunes Nesi , Wagner L. Araújo , and Hans‐Peter Braun . 2015. “Amino Acid Catabolism in Plants.” Molecular plant 8: 1563–1579. 10.1016/j.molp.2015.09.005 26384576

[50] Galili, Gad , Tamar Avin‐Wittenberg , Ruthie Angelovici , and Alisdair R. Fernie . 2014. “The Role of Photosynthesis and Amino Acid Metabolism in the Energy Status During Seed Development.” Frontiers in Plant Science 5: 447. 10.3389/fpls.2014.00447 PMC4153028 25232362

[51] Rojas, Clemencia M. , Muthappa Senthil‐Kumar , Vered Tzin , and Kirankumar S. Mysore . 2014. “Regulation of Primary Plant Metabolism During Plant‐Pathogen Interactions and Its Contribution to Plant Defense.” Frontiers in Plant Science 5: 17. 10.3389/fpls.2014.00017 PMC3919437 24575102

[52] Yun, Ze , Feng Zhu , Ping Liu , Yunliu Zeng , Juan Xu , Yunjiang Cheng , and Xiuxin Deng . 2015. “Sweating Treatment Enhances Citrus Fruit Disease Resistance By Inducing the Accumulation of Amino Acids and Salicylic Acid‐Induced Resistance Pathway.” Physiologia Plantarum 155: 109–125. 10.1111/ppl.12340 25893482

[53] Berger, S. , A. K. Sinha , and T. Roitsch . 2007. “Plant Physiology Meets Phytopathology: Plant Primary Metabolism and Plant–Pathogen Interactions.” Journal of Experimental Botany 58: 4019–4026. 10.1093/jxb/erm298 18182420

[54] Tzin, Vered , and Gad Galili . 2010. “New Insights into the Shikimate and Aromatic Amino Acids Biosynthesis Pathways in Plants.” Molecular plant 3: 956–972. 10.1093/mp/ssq048 20817774

[55] Wang, Zhan Qi , Xiao Yan Xu , Qiao Qiao Gong , Chen Xie , Wei Fan , Jian Li Yang , Qi Shan Lin , and Shao Jian Zheng . 2014. “Root Proteome of Rice Studied by iTRAQ Provides Integrated Insight Into Aluminum Stress Tolerance Mechanisms in Plants.” Journal of Proteomics 98: 189–205. 10.1016/j.jprot.2013.12.023 24412201

[56] Munir, Nigarish , Chunzhen Cheng , Chaoshui Xia , Xuming Xu , Muhammad Azher Nawaz , Junaid Iftikhar , Yukun Chen , Yuling Lin , and Zhongxiong Lai . 2019. “RNA‐Seq Analysis Reveals an Essential Role of Tyrosine Metabolism Pathway in Response to Root‐Rot Infection in Gerbera Hybrida.” PloS One 14: e0223519. 10.1371/journal.pone.0223519 PMC6808435 31644543

[57] Zhang, Xuebin , and Chang‐Jun Liu . 2015. “Multifaceted Regulations of Gateway Enzyme Phenylalanine Ammonia‐Lyase in the Biosynthesis of Phenylpropanoids.” Molecular Plant 8: 17–27. 10.1016/j.molp.2014.11.001 25578269

[58] Noel, Joseph P. , Michael B. Austin , and Erin K. Bomati . 2005. “Structure–Function Relationships in Plant Phenylpropanoid Biosynthesis.” Current Opinion in Plant Biology 8: 249–253. 10.1016/j.pbi.2005.03.013 PMC2861907 15860421

[59] Zhao, Shicheng , Pham Anh Tuan , Xiaohua Li , Yeon Bok Kim , HyeRan Kim , Chun Geon Park , Jingli Yang , Cheng Hao Li , and Sang Un Park . 2013. “Identification of Phenylpropanoid Biosynthetic Genes and Phenylpropanoid Accumulation By Transcriptome Analysis of Lycium chinense.” BMC Genomics 14: 802. 10.1186/1471-2164-14-802 PMC4046672 24252158

[60] Tetreault, Hannah M. , Tammy Gries , Nathan A. Palmer , Deanna L. Funnell‐Harris , Shirley Sato , Zhengxiang Ge , Gautam Sarath , and Scott E. Sattler . 2020. “Overexpression of Ferulate 5‐hydroxylase Increases Syringyl Units in Sorghum bicolor.” Plant Molecular Biology 103: 269–285. 10.1007/s11103-020-00991-3 32170550

[61] Nitzsche, Richard , Özlem Günay‐Esiyok , Maximilian Tischer , Vyacheslav Zagoriy , and Nishith Gupta . 2017. “A Plant/Fungal‐Type Phosphoenolpyruvate Carboxykinase Located in the Parasite Mitochondrion Ensures Glucose‐Independent Survival of Toxoplasma gondii.” Journal of Biological Chemistry 292: 15225–15239. 10.1074/jbc.M117.802702 PMC5602384 28726641

[62] Nguyen, Hoa Thanh , Lingyun Li , Akifumi Eguchi , Tetsuro Agusa , Kimika Yamamoto , Kurunthachalam Kannan , Eun‐Young Kim , and Hisato Iwata . 2022. “Effects of Gestational Exposure to Bisphenol A on the Hepatic Transcriptome and Lipidome of Rat Dams: Intergenerational Comparison of Effects in the Offspring.” Science of the Total Environment 826: 153990. 10.1016/j.scitotenv.2022.153990 35192832

[63] Yang, Liyang , Soon‐Woong Chang , Hyun‐Sang Shin , and Jin Hur . 2015. “Tracking the Evolution of Stream DOM Source During Storm Events Using End Member Mixing Analysis Based on DOM Quality.” Journal of Hydrology 523: 333–341. 10.1016/j.jhydrol.2015.01.074

[64] Wu, Dongming , Changqi Ren , Dong Ren , Yingjie Tian , Youping Li , Chunyuan Wu , and Qinfen Li . 2023. “New Insights into Carbon Mineralization in Tropical Paddy Soil Under Land Use Conversion: Coupled Roles of Soil Microbial Community, Metabolism, and Dissolved Organic Matter Chemodiversity.” Geoderma 432: 116393. 10.1016/j.geoderma.2023.116393

[65] Eichmann, Ruth , Luke Richards , and Patrick Schäfer . 2021. “Hormones As Go‐Betweens in Plant Microbiome Assembly.” The Plant Journal 105: 518–541. 10.1111/tpj.15135 PMC8629125 33332645

[66] EL Sabagh, Ayman , Mohammad Sohidul Islam , Akbar Hossain , Muhammad Aamir Iqbal , Muhammad Mubeen , Mirza Waleed , Mariana Reginato , et al. 2022. “Phytohormones As Growth Regulators During Abiotic Stress Tolerance in Plants.” Frontiers in Agronomy 4: 765068. 10.3389/fagro.2022.765068

[67] Ansari, Firoz Ahmad , Iqbal Ahmad , and John Pichtel . 2019. “Growth Stimulation and Alleviation of Salinity Stress to Wheat By the Biofilm Forming Bacillus pumilus strain FAB10.” Applied Soil Ecology 143: 45–54. 10.1016/j.apsoil.2019.05.023

[68] Plastics‐The Facts 2023: An Analysis of European Latest Plastics Production, Demand and Waste Data (last accessed) https://plasticseurope.org/knowledge-hub/plastics-the-fast-facts-2023/

[69] Fuller, Stephen , and Anil Gautam . 2016. “A Procedure for Measuring Microplastics Using Pressurized Fluid Extraction.” Environmental Science & Technology 50: 5774–5780. 10.1021/acs.est.6b00816 27172172

[70] Hill, Micheal W. , Bryan G. Hopkins , and Von D. Jolley . 2015. “Maize In‐Season Growth Response to Organic Acid‐Bonded Phosphorus Fertilizer (Carbond P®).” Journal of Plant Nutrition 38: 1398–1415. 10.1080/01904167.2014.973040

[71] Shah, Anis Ali , Waheed Ullah Khan , Nasim Ahmad Yasin , Waheed Akram , Aqeel Ahmad , Muhammad Abbas , Aamir Ali , and Muhammad Naeem Safdar . 2020. “Butanolide Alleviated Cadmium Stress By Improving Plant Growth, Photosynthetic Parameters and Antioxidant Defense System of *Brassica Oleracea* .” Chemosphere 261: 127728. 10.1016/j.chemosphere.2020.127728 32731022

[72] Kim, Daehwan , Geo Pertea , Cole Trapnell , Harold Pimentel , Ryan Kelley , and Steven L. Salzberg . 2013. “TopHat2: Accurate Alignment of Transcriptomes in the Presence of Insertions, Deletions and Gene Fusions.” Genome Biology 14: R36. 10.1186/gb-2013-14-4-r36 PMC4053844 23618408

[73] Pertea, Mihaela , Geo M. Pertea , Corina M. Antonescu , Tsung‐Cheng Chang , Joshua T. Mendell , and Steven L. Salzberg . 2015. “StringTie Enables Improved Reconstruction of a Transcriptome From RNA‐seq Reads.” Nature Biotechnology 33: 290–295. 10.1038/nbt.3122 PMC4643835 25690850

[74] Li, Bo , and Colin N. Dewey . 2011. “RSEM: Accurate Transcript Quantification From RNA‐Seq Data With or Without a Reference Genome.” BMC Bioinformatics 12: 323. 10.1186/1471-2105-12-323 PMC3163565 21816040

[75] Genovese, Christopher R. , Nicole A. Lazar , and Thomas Nichols . 2002. “Thresholding of Statistical Maps in Functional Neuroimaging Using the False Discovery Rate.” Neuroimage 15: 870–878. 10.1006/nimg.2001.1037 11906227

[76] Wickham, H. 2009. ggplot2: Elegant Graphics for Data Analysis. 10.1007/978-0-387-98141-3

