# Supplementary material for: Transcriptomic and metabolomic responses of maize under conventional and biodegradable microplastic stress
Source: IMetaOmics. 2024 Dec 28;2(1):e48. doi: 10.1002/imo2.48 (PMC12806533; doi:10.1002/imo2.48)
Supplement: Supplementary file 1 — Figure S1. The fourier transform infrared spectroscopy (FTIR) results of the five microplastics particles. Figure S2. The scanning electron microscopy (SEM) photos of the five microplastic particles. Figure S3. Responses of antioxidant defense system and photosynthetic pigment of maize to microplastic. Figure S4. Mantel test analyzed the relationship between the phenotypic changes of maize and antioxidant and photosynthetic index in (A) all treatments, (B) biodegradable microplastic treatments, (C) conventional microplastic treatment. Figure S5. Volcano plot of differentially expressed genes (DEGs) in maize between the control and microplastic treatments. Figure S6. Kyoto Encyclopedia of Genes and Genomes (KEGG) enrichment analysis of DEGs in different treatments. Figure S7. Venn diagram of DEGs in different microplastics treatments (A). KEGG pathway analysis of unique DEGs in different microplastics treatments (B). Figure S8. PTL, ERF1, HSP20, and GAPDH gene expression in different treatments and the control. Figure S9. The metabolites contributing to separation along with Component 1 and 2 based on sparse partial least‐squares discriminant analysis (sPLS‐DA) (A), and the functional pathways of the biomarker metabolites (B). Figure S10. Volcano plot of differentially expressed metabolites (DEMs) in maize between the control and microplastic treatments. Figure S11. KEGG enrichment analysis of DEMs in different treatments. Figure S12. Transcription−metabolism regulation integration network of enriched pathways in different treatments. Figure S13. DIABLO analysis demonstrated the relationship between antioxidant and photosynthetic parameters, representative pathway genes, and metabolites. Figure S14. The DOM properties in soil under different treatments. Figure S15. The bacterial communities in soil under different treatments. [file IMO2-2-e48-s002.docx]

**Supporting information for:**

**Transcriptomic and Metabolomic Responses of Maize under Conventional and Biodegradable Microplastic Stress**

**Running title: transcriptomic and metabolomic responses of maize under microplastic stress**

Yuanze Sun^1^, Jinxi Zang^1^, Siyuan Xie^1^, Mochen Wu^1^, Jianguo Tao^1^, Tanveer M. Adyel^2^, Xinyu Du^3^, Si Li^1^, Jie Wang^1*^

^1^ Beijing Key Laboratory of Farmland Soil Pollution Prevention and Remediation, College of Resources and Environmental Sciences, China Agricultural University, Beijing 100193, China

^2^ Biosciences and Food Technology Discipline, RMIT University, Melbourne, VIC 3000, 26 Australia

^3^ Collage of Maine Ecology and Environment, Shanghai Ocean University, Shanghai 201306, China

^*^Correspondence: [jiewangcau@cau.edu.cn](mailto:jiewangcau@cau.edu.cn) (Jie Wang)

Summary

Number of pages: 25

Number of tables: 5

Number of figures: 15

Content

Text S1 Estimation of photosynthetic parameters.

Text S2 Metabolomic Data Processing and Analysis.

Text S3 Dissolved organic matter (DOM) Fluorescent Calculation.

Text S4 Amplification Process and Bioinformation Analysis.

Figure S1. The fourier transform infrared spectroscopy (FTIR) results of the five microplastics particles.

Figure S2. The scanning electron microscopy (SEM) photos of the five microplastic particles.

Figure S3. Responses of antioxidant defense system and photosynthetic pigment of maize to microplastic. Activities of peroxidase (POD), polyphenol oxidase (PPO), ascorbate peroxidase (APX), and glutathione S-transferase (GST), and contents of chlorophyll a, chlorophyll b, and total chlorophyll in different treatments. Different letters denoted significant differences at 0.05 probability levels. Vertical bar represents standard error ± SE among four replicates.

Figure S4. Mantel test analyzed the relationship between the phenotypic changes of maize (chlall, chlA, and chlB) and antioxidant (glutathione (GSH), abscisic acid (ABA), POD, glutathione S-transferase (CAT), glutathione reductase (GR) , and ascorbic acid (ASA), GST, PPO, APX, malondialdehyde (MDA)) and photosynthetic index (stomatal limitation (Ls), transpiration rate (Tr), intercellular carbon dioxide (Ci), stomatal conductance (Gs), and photosynthetic rate (Pn)) in (A) all treatments, (B) biodegradable microplastic treatments, (C) conventional microplastic treatment. *, **, and *** indicate the significant level at *p* < 0.05, p < 0.01, and p < 0.001 in figures.

Figure S5. Volcano plot of differentially expressed genes (DEGs) in maize between the control and microplastic treatments.

Figure S6. Kyoto Encyclopedia of Genes and Genomes (KEGG) enrichment analysis of DEGs in different treatments. The ordinate represents the classification of KEGG. The abscissa is the rich factor which means the ratio of the number of DEGs in the pathway to the total number of genes in the pathway. The size of the dot indicates the number of genes in the pathway. The bigger the spot, the more the number of DEGs. The closer the *p* value is to 0, the more significant the enrichment is.

Figure S7. Venn diagram of DEGs in different microplastics treatments (A). KEGG pathway analysis of unique DEGs in different microplastics treatments (B).

Figure S8. *PTL*, *ERF1*, *HSP20*, and *GAPDH* gene expression in different treatments and the control. *, **, and *** indicate the significant level at *p* < 0.05, *p* < 0.01, and *p* < 0.001 in figures.

Figure S9. The metabolites contributing to separation along with Component 1 and 2 based on sparse partial least-squares discriminant analysis (sPLS-DA) (A), and the functional pathways of the biomarker metabolites (B).

Figure S10. Volcano plot of differentially expressed metabolites (DEMs) in maize between the control and microplastic treatments.

Figure S11. KEGG enrichment analysis of DEMs in different treatments. The abscissa represents the classification of KEGG. The ordinate represents the rich factor which means the ratio of the number of DEMs in the pathway to the total number of genes in the pathway. The closer the *p* value is to 0, the more significant the enrichment is.

Figure S12. Transcription−metabolism regulation integration network of enriched pathways in different treatments. Nodes represent functional pathways; edges represent the correlations in different microplastic treatments. The node color indicates its degree.

Figure S13. DIABLO analysis demonstrated the relationship between antioxidant and photosynthetic parameters, representative pathway genes, and metabolites.

Figure S14. The DOM properties in soil under different treatments. Biological index (BIX), humification index (HIX), and fluorescent index (FI) of DOM in different treatments (A). Principal component analysis of the distribution of DOM fluorescent characteristics among different treatments (B). Relative distribution of fluorescent components (C1−C5) calculated by PARAFAC modeling in different treatments (C).

Figure S15. The bacterial communities in soil under different treatments. The richness, diversity, and evenness of bacterial community in different treatments (A). The principal coordinates analysis (PCoA) of bacterial communities in different treatments (B). The relative abundance of the main phyla in different treatments (C).

Table S1. The physicochemical properties of soil used in this study.

Table S2. Elution gradient of mobile phase that used for UPLC-MS/MS analyses. Solvent A: aqueous formic acid (1% (v/v) formic acid). Solvent B: acetonitrile/isopropanol (1/1, v/v) (0.1% (v/v) formic acid).

Table S3. Photosynthetic parameters of maize in different treatments. The asterisk (*) indicates a statistically significant difference at *p* < 0.05 compared with the control and microplastic treatments.

Table S4. Soil properties under different treatments. (electrical conductivity, EC; dissolved organic carbon, DOC; ammonium, NH_4_^+^-N; nitrate, NO_3_^-^-N; and available phosphorus, AP). Different letters indicate statistically significant differences among treatments (*p* < 0.05).

Table S5. Description of five component parallel factor analysis (PARAFAC) model.

Text S1 Estimation of photosynthetic parameters

Leaf gas exchange parameters of photosynthetic rate (Pn, µmol (CO_2_) m^–2^ s^–1^), stomatal conductance (Gs, mol (H_2_O) m^−2^ s^−1^), intercellular CO_2_ concentration (Ci, μmol CO_2_ mol^-1^), transpiration rate (Tr, mmol (H_2_O) m^–2^ s^–1^) and the ratio of intercellular CO_2_ concentration/ambient CO_2_ (Ci/Ca) were measured under different treatments. Based on the photosynthetic parameters, the values of stomatal limitation (L_s_), leaf intrinsic water use efficiency (LWUE_int_) and leaf instantaneous water use efficiency (LWUE_ins_) were calculated according to the following equations [1]:

Ls = 1 ﹣ $\frac{Ci}{Ca}$

LWUE_int_ = $\frac{Pn}{\mathrm{Tr}}$

LWUE_ins_ = $\frac{Pn}{\mathrm{Gs}}$

Text S2 Metabolomic Data Processing and Analysis

UPLC-MS/MS raw data was imported into metabolomics software Progenesis Qi (Waters Corporation, Milford, USA). The MS data such as peak extraction, peak matching, and peak alignment were preprocessed in Progenesis Qi. The data were normalized by total ionic strength. The quality control (QC) samples of all samples were taken as a small sample group and injected at the beginning, periodic interval, and the end of the sample queue under the same chromatographic condition. Metabolic characteristics whose QC was greater than 30% relative standard deviation (RSD) were discarded. The quality data were collected to evaluate the sensitivity and stability of the instrument performance based on the quality accuracy, retention time stability, and variation coefficient. Finally, the data matrices, including retention time, and peak intensity, were obtained [2]. Identification of metabolites was based on the primary and secondary spectral data annotated against public databases, namely HMDB (<http://www.hmdb.ca/>) and METLIN (<http://metlin.scripps.edu/index.php>). The obtained results were subjected to sparse partial least-squares discriminant analysis (sPLS-DA) conducted using the R package mixOmicsx. DESeq2 R package (1.40.2) was used to identify the differentially expressed metabolites (DEMs) by |log_2_-fold change| >1 and *p* adjust-value < 0.05. The p values were adjusted using the Benjamini and Hochberg’s approach for controlling the false discovery rate (FDR) [3].

Text S3 Dissolved organic matter (DOM) Fluorescent Calculation

Fluorescence measurements were conducted using an Aqualog Analyzer (HORIBA Scientific, USA). Samples were diluted to avoid inner filter effects by using Milli-Q water. The scan program was set as follows: the emission spectra from 280 to 600 nm at 2 nm intervals and increasing excitation wavelength from 250 to 550 nm at 5 nm increments. The measurement was conducted at room temperature, and the instrument bias was corrected with a correction file provided by the manufacturer. Subsequently, EEM data were blank-subtracted, and the first and second Rayleigh and Raman peaks were removed. Raman normalization was performed according to the method described by Murphy et al. Three fluorescent indices, including fluorescence index (FI), humic index (HIX), and biological index (BIX), were calculated based on the EEM spectra. FI was determined as the ratio of emission intensity at 450 nm to that at 500 nm, measured at an excitation wavelength of 370 nm [4]. HIX was calculated as the ratio of the peak area under the emission spectra 435 – 480 nm to that under 300 – 345 nm plus 435 – 480 nm, at an excitation wavelength of 254 nm [5, 6]. BIX was computed as the ratio of emission intensity at 380 nm to that at 430 nm, obtained at an excitation wavelength of 310 nm [7, 8]. The parallel factor analysis (PARAFAC) model was employed to analyze the excitation-emission matrix (EEM) fluorescence spectral data. To conduct the PARAFAC analysis, a three-dimensional dataset—comprising excitation wavelength, emission wavelength, and sample numbers—was constructed [9]. The modeling process and visualization were performed using the R package "StaRdom," following the instructions provided by Murphy et al [10].

Text S4 Amplification Process and Bioinformation Analysis.

Soil DNA samples were extracted using DNeasy PowerSoil Kit (Qiagen, Shanghai, China), following the manufacturer’s instructions. The quality and concentration of the isolated DNA were assessed using a NanoDrop ND2000 spectrophotometer (NanoDrop Technologies, USA). The bacterial V3 – V4 regions of the 16S rRNA gene were amplified using the 338F (ACTCCTACGGGAGGCAGCA) and 806R (GGACTACHVGGGTWTCTAAT) primers. The PCR reactions were carried out in 25 μL reactions with 2.5 μL 10 × PCR buffer II, 0.5 unit of AccuPrime Taq DNA Polymerase High Fidelity (Invitrogen, Carlsbad, CA, USA), 0.4 μM of each primer, and 10 ng of template DNA. The PCR cycling was performed following 3 min of denaturation at 95 ℃, 27 cycles of 30 s at 95 ℃, 30 s at 55 ℃, and 45 s at 72 ℃, and a final extension at 72 ℃ for 10 min. After purification, the amplicons were sequenced in a 2 × 300 bp paired-end format using the Illumina MiSeq platform by Majorbio BioPharm Technology Co. Ltd (Shanghai, China). Sequence processing and analyses were performed using the open-source platform QIIME2. Sequence qualities were evaluated, and paired-end sequences were merged. Raw sequences were demultiplexed, and then trimmed and quality controlled by using the DADA2 plugin in QIIME2. Following quality control, the denoised reads were clustered into amplicon sequence variants (ASVs), and taxonomic information was assigned to each representative ASV using the Silva 138 database.


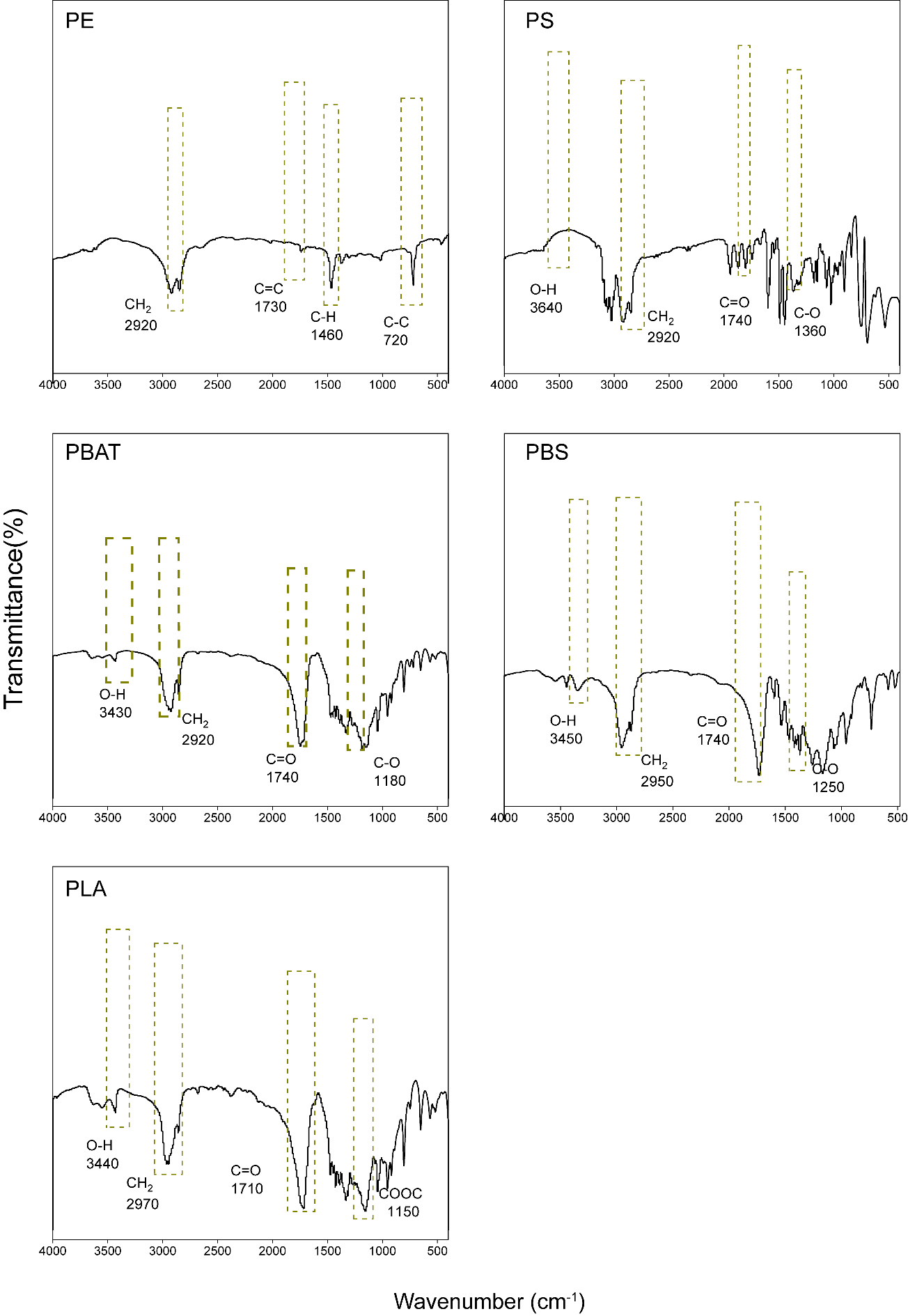


Figure S1. The fourier transform infrared spectroscopy (FTIR) results of the five microplastics particles.


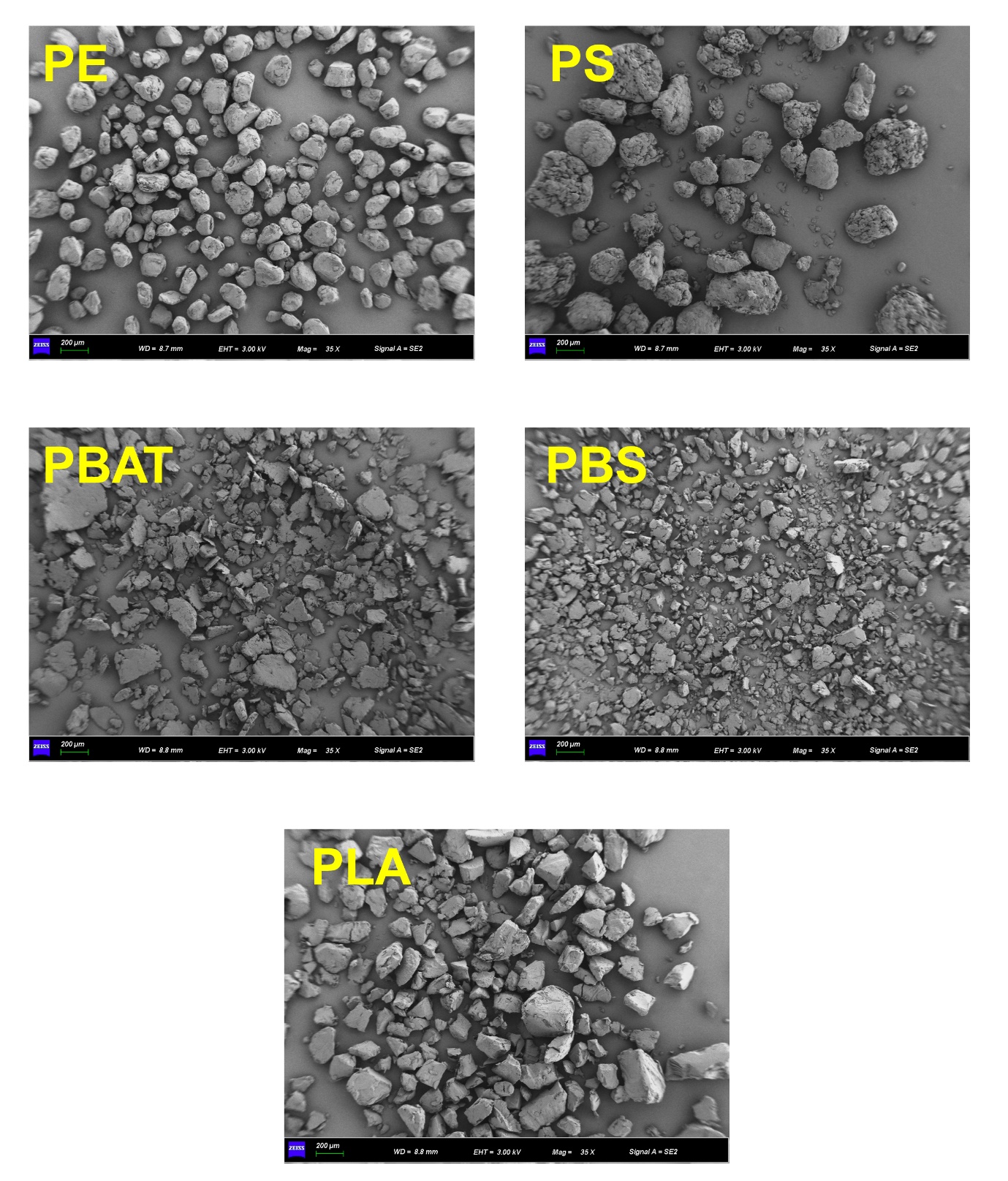


Figure S2. The scanning electron microscopy (SEM) photos of the five microplastic particles.

**
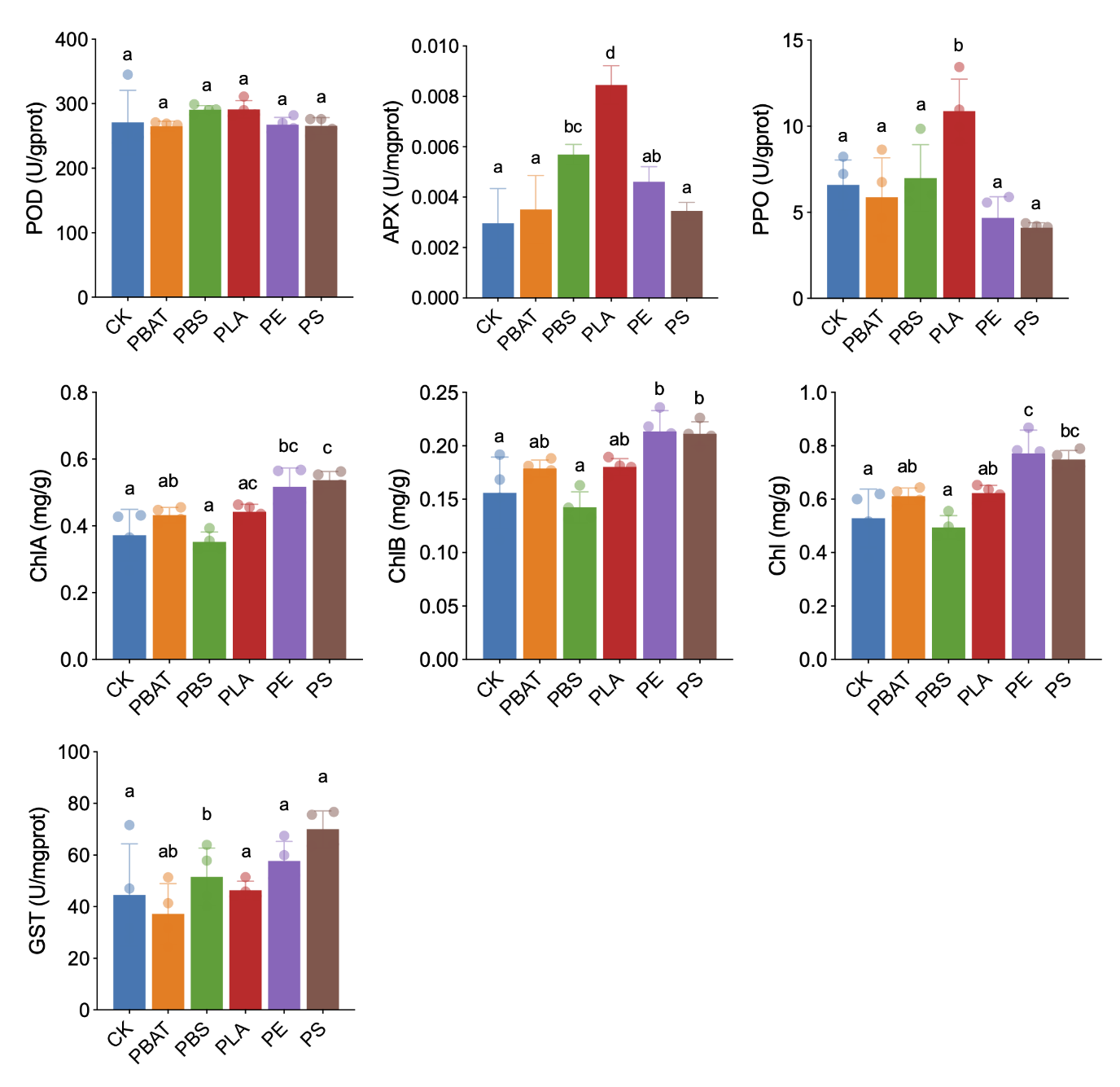
**

Figure S3. Responses of antioxidant defense system and photosynthetic pigment of maize to microplastic. Activities of peroxidase (POD), polyphenol oxidase (PPO), ascorbate peroxidase (APX), and glutathione S-transferase (GST), and contents of chlorophyll a (ChlA), chlorophyll b (ChlB), and total chlorophyll (Chl) in different treatments. Different letters denoted significant differences at 0.05 probability levels. Vertical bar represents standard error ± SE among four replicates.

Figure S4. Mantel test analyzed the relationship between the phenotypic changes of maize (chlall, chlA, and chlB) and antioxidant (glutathione (GSH), abscisic acid (ABA), POD, glutathione S-transferase (CAT), glutathione reductase (GR) , and ascorbic acid (ASA), GST, PPO, APX, malondialdehyde (MDA)) and photosynthetic index (stomatal limitation (Ls), transpiration rate (Tr), intercellular carbon dioxide (Ci), stomatal conductance (Gs), and photosynthetic rate (Pn)) in (A) all treatments, (B) biodegradable microplastic treatments, (C) conventional microplastic treatment. *, **, and *** indicate the significant level at *p* < 0.05, *p* < 0.01, and *p* < 0.001 in figures.

Figure S5. Volcano plot of differentially expressed genes (DEGs) in maize between the control and microplastic treatments.

Figure S6. Kyoto Encyclopedia of Genes and Genomes (KEGG) enrichment analysis of DEGs in different treatments. The ordinate represents the classification of KEGG. The abscissa is the rich factor which means the ratio of the number of DEGs in the pathway to the total number of genes in the pathway. The size of the dot indicates the number of genes in the pathway. The bigger the spot, the more the number of DEGs. The closer the *p* value is to 0, the more significant the enrichment is.


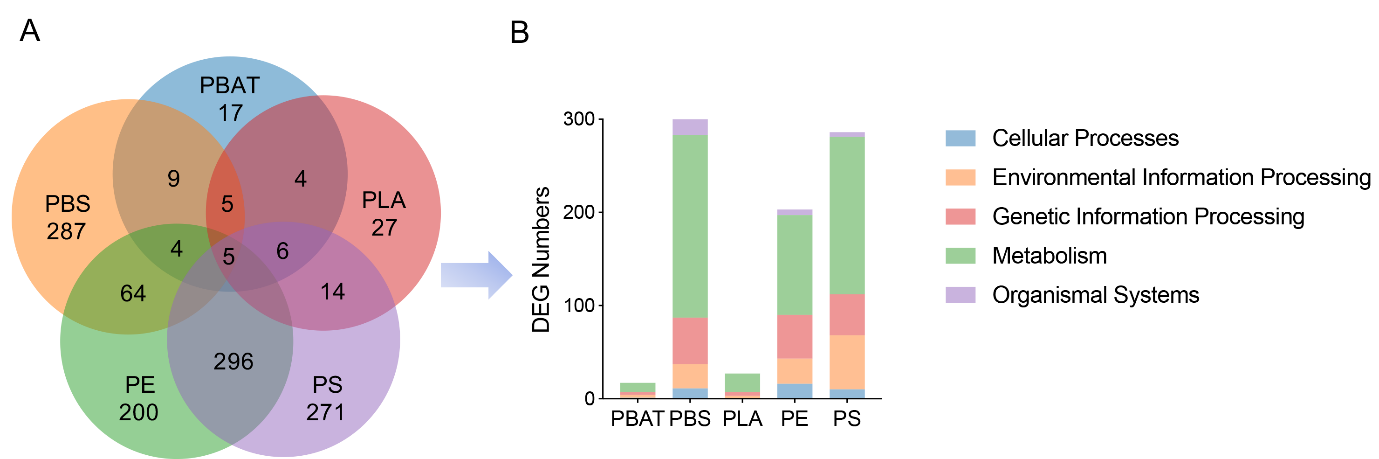


Figure S7. Venn diagram of DEGs in different microplastics treatments (A). KEGG pathway analysis of unique DEGs in different microplastics treatments (B).


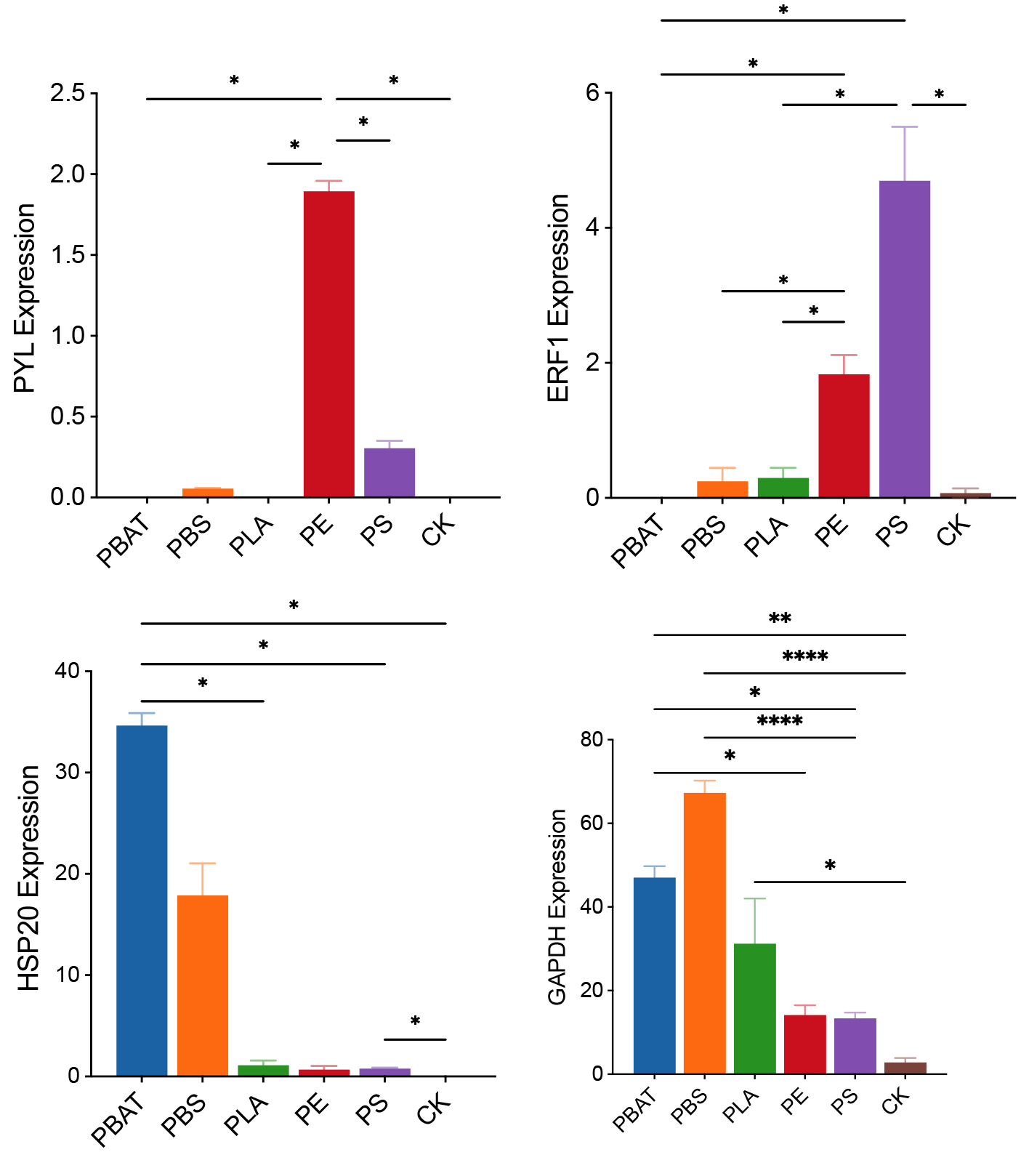


Figure S8. *PTL*, *ERF1*, *HSP20*, and *GAPDH* gene expression in different treatments and the control. *, **, and *** indicate the significant level at *p* < 0.05, *p* < 0.01, and *p* < 0.001 in figures.


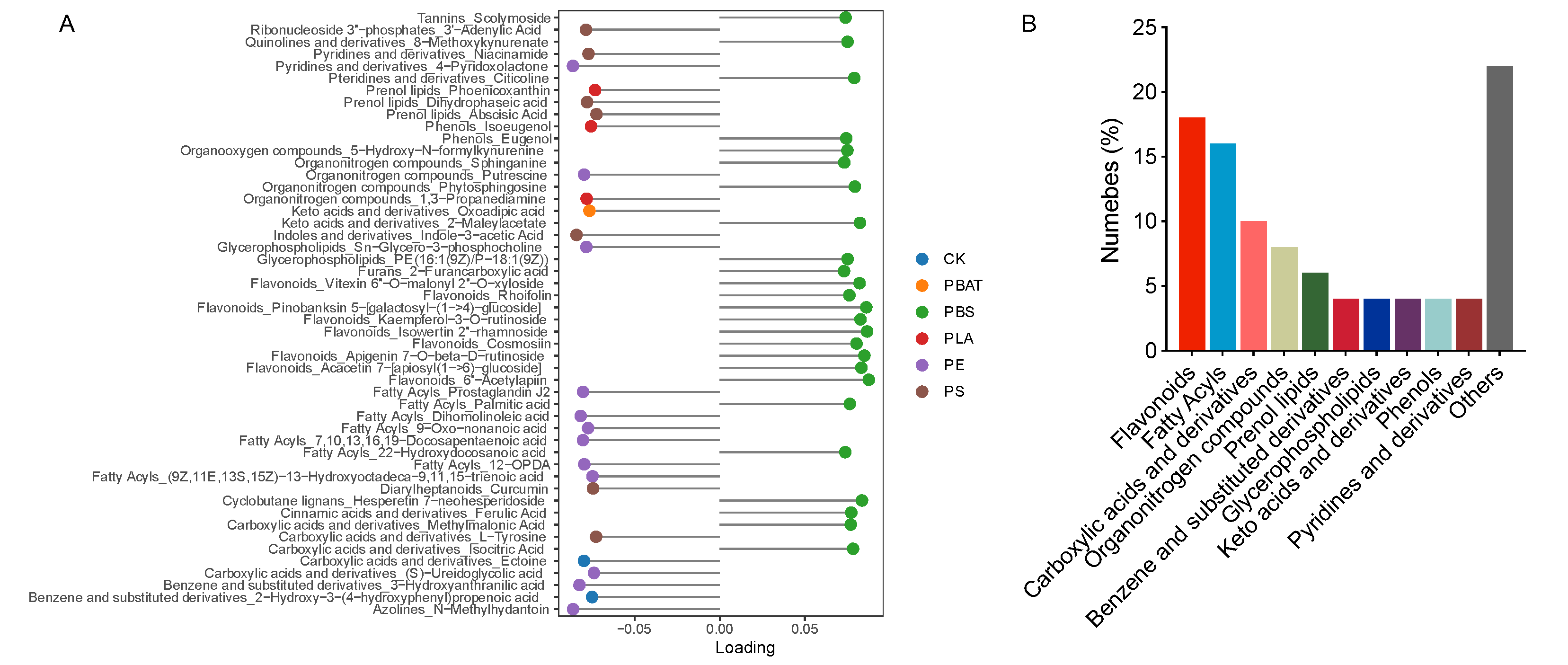


Figure S9. The metabolites contributing to separation along with Component 1 and 2 based on sparse partial least-squares discriminant analysis (sPLS-DA) (A), and the functional pathways of the biomarker metabolites (B).

Figure S10. Volcano plot of differentially expressed metabolites (DEMs) in maize between the control and microplastic treatments.

Figure S11. KEGG enrichment analysis of DEMs in different treatments. The abscissa represents the classification of KEGG. The ordinate represents the rich factor which means the ratio of the number of DEMs in the pathway to the total number of genes in the pathway. The closer the *p* value is to 0, the more significant the enrichment is.


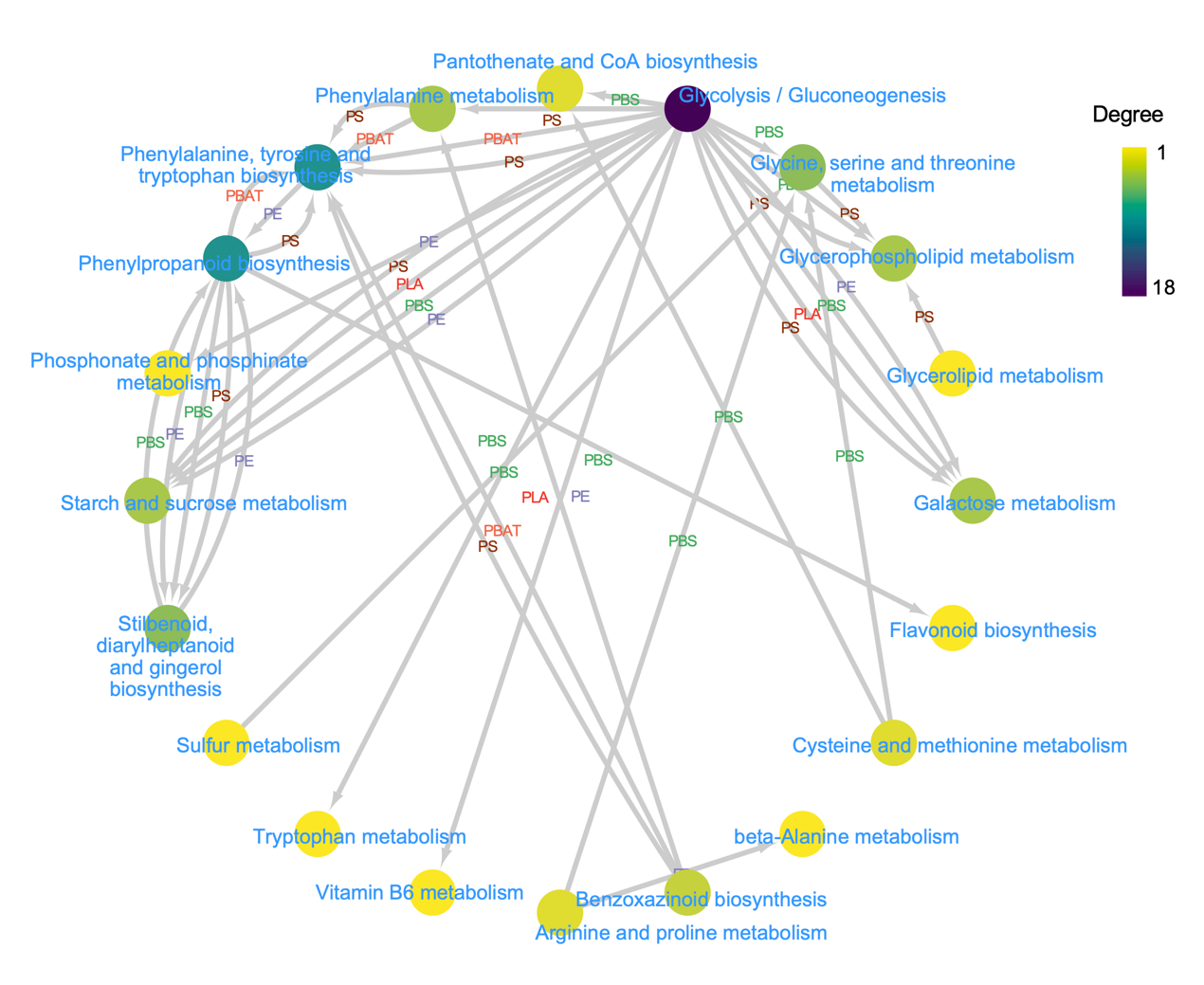


Figure S12. Transcription−metabolism regulation integration network of enriched pathways in different treatments. Nodes represent functional pathways; edges represent the correlations in different microplastic treatments. The node color indicates its degree.

Figure S13. DIABLO analysis demonstrated the relationship between antioxidant and photosynthetic parameters, representative pathway genes, and metabolites.


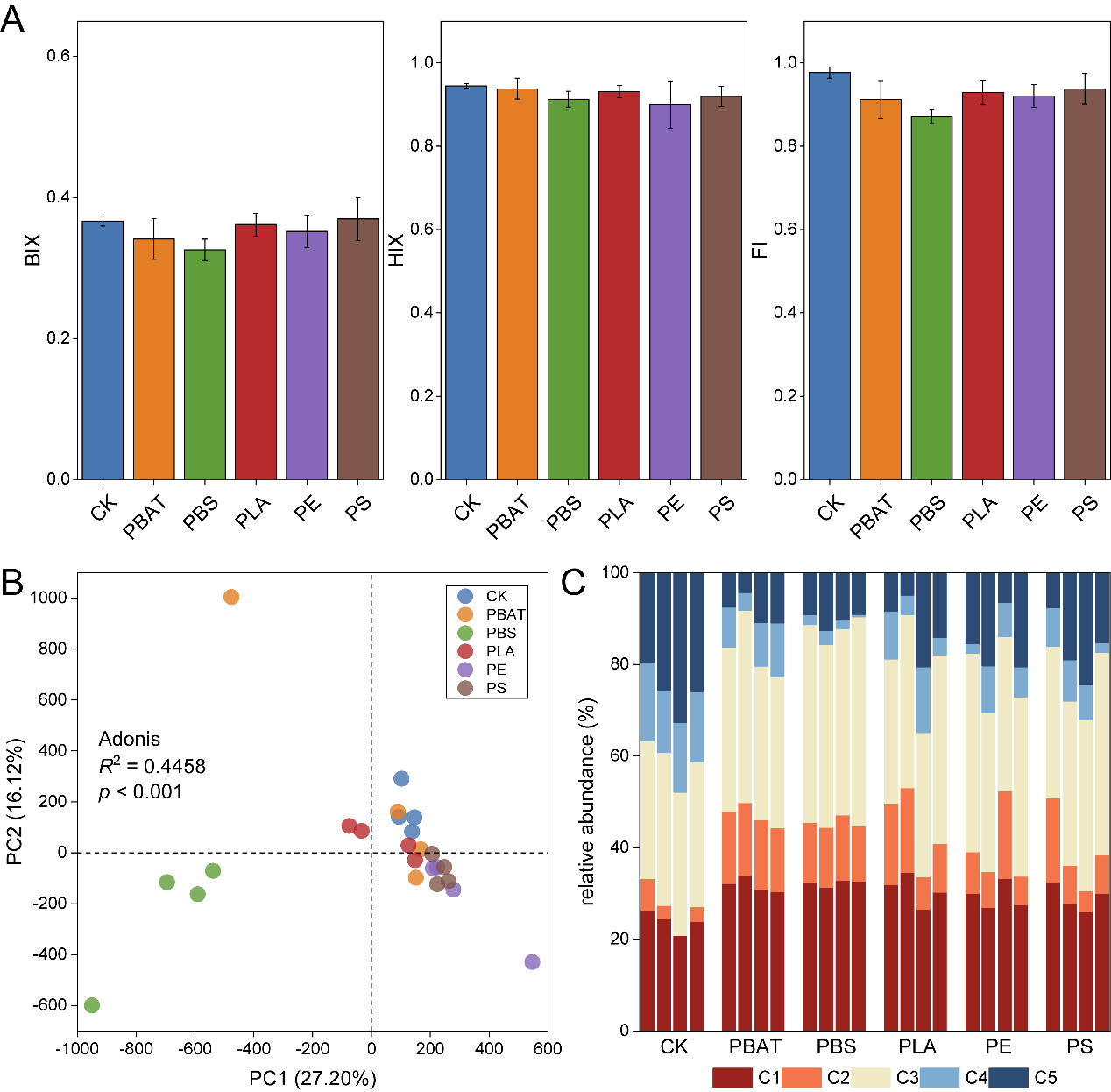


Figure S14. The DOM properties in soil under different treatments. Biological index (BIX), humification index (HIX), and fluorescent index (FI) of DOM in different treatments (A). Principal component analysis of the distribution of DOM fluorescent characteristics among different treatments (B). Relative distribution of fluorescent components (C1−C5) calculated by PARAFAC modeling in different treatments (C).


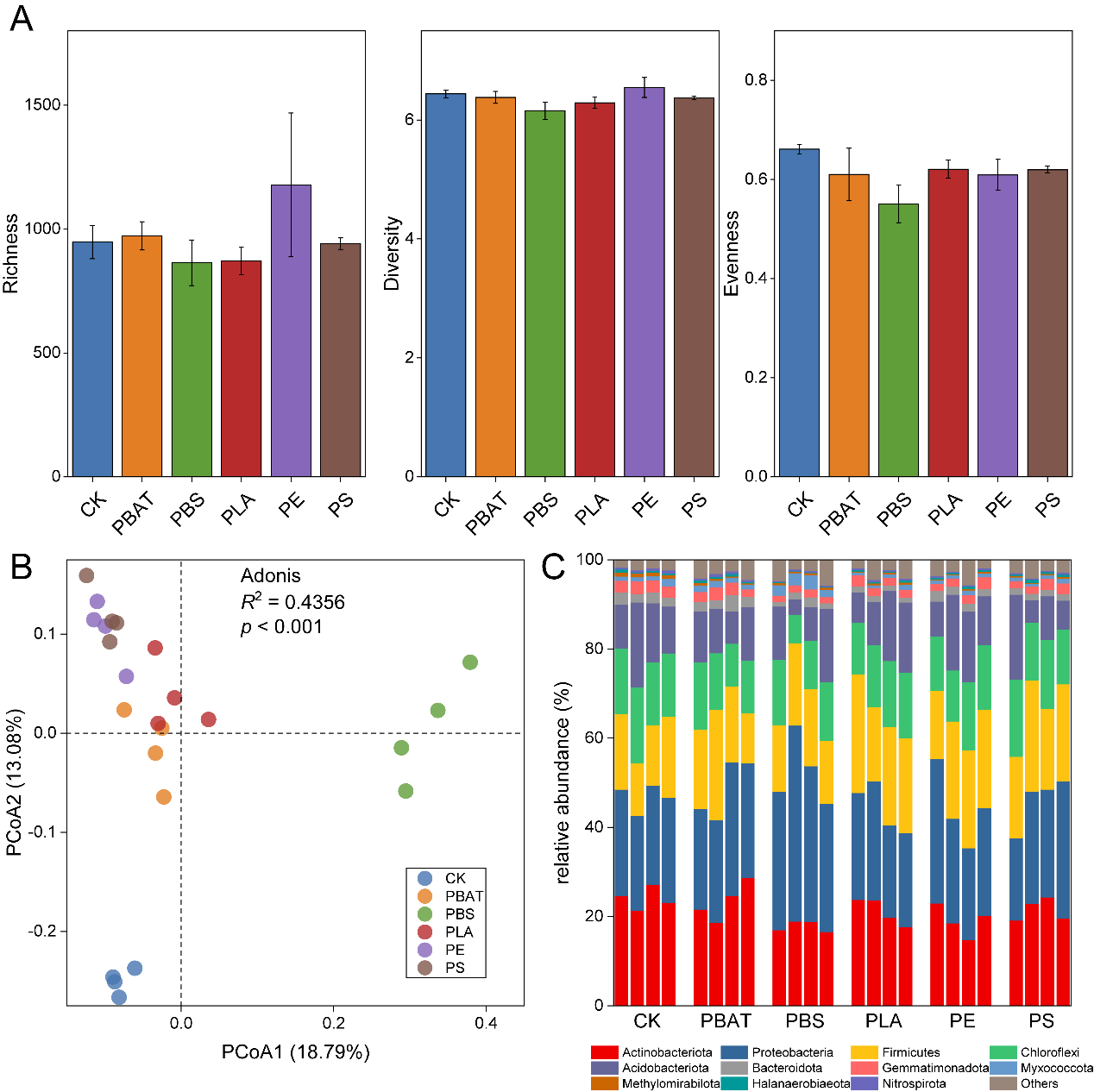


Figure S15. The bacterial communities in soil under different treatments. The richness, diversity, and evenness of bacterial community in different treatments (A). The principal coordinates analysis (PCoA) of bacterial communities in different treatments (B). The relative abundance of the main phyla in different treatments (C).

1. Liu, Jie, Hui Li, Zhenyu Yuan, Jiajia Feng, Shuaihong Chen, Guangzhao Sun, Zhenhua Wei, Tiantian Hu. 2024. “Effects of microbial fertilizer and irrigation amount on growth, physiology and water use efficiency of tomato in greenhouse.” *Scientia Horticulturae* 323: 112553. <https://doi.org/10.1016/j.scienta.2023.112553>

2. Kang, Min, Hongxia Wang, Chuxin Chen, Ran Suo, Jianfeng Sun, Quanhong Yue, Yaqiong Liu. 2023. “Analytical strategies based on untargeted and targeted metabolomics for the accurate authentication of organic milk from Jersey and Yak.” *Food Chemistry: X* 19: 100786. <https://doi.org/10.1016/j.fochx.2023.100786>

3. Genovese, Christopher R., Nicole A. Lazar, Thomas Nichols. 2002. “Thresholding of Statistical Maps in Functional Neuroimaging Using the False Discovery Rate.” *Neuroimage* 15: 870-878. <https://doi.org/10.1006/nimg.2001.1037>

4. McKnight, Diane M, Elizabeth W Boyer, Paul K Westerhoff, Peter T Doran, Thomas Kulbe, Dale T Andersen. 2001. “Spectrofluorometric characterization of dissolved organic matter for indication of precursor organic material and aromaticity.” *Limnology and Oceanography* 46: 38-48. <https://doi.org/10.4319/lo.2001.46.1.0038>

5. Zsolnay, Adam, Erik Baigar, Miguel Jimenez, Bernd Steinweg, Flavia Saccomandi. 1999. “Differentiating with fluorescence spectroscopy the sources of dissolved organic matter in soils subjected to drying.” *Chemosphere* 38: 45-50. <https://doi.org/10.1016/S0045-6535(98)00166-0>

6. Ohno, Tsutomu. 2002. “Fluorescence inner-filtering correction for determining the humification index of dissolved organic matter.” *Environmental Science & Technology* 36: 742-746. <https://doi.org/10.1021/es0155276>

7. Huguet, Arnaud, Lilian Vacher, Stéphane Relexans, Sylvain Saubusse, Jean-Marie Froidefond, Edith Parlanti. 2009. “Properties of fluorescent dissolved organic matter in the Gironde Estuary.” *Organic Geochemistry* 40: 706-719. <https://doi.org/10.1016/j.orggeochem.2009.03.002>

8. Parlanti, Edith, K Wörz, Laure Geoffroy, Michel Lamotte. 2000. “Dissolved organic matter fluorescence spectroscopy as a tool to estimate biological activity in a coastal zone submitted to anthropogenic inputs.” *Organic Geochemistry* 31: 1765-1781. <https://doi.org/10.1016/S0146-6380(00)00124-8>

9. Murphy, Kathleen R, Colin A Stedmon, Daniel Graeber, Rasmus Bro. 2013. “Fluorescence spectroscopy and multi-way techniques. PARAFAC.” *Analytical Methods* 5: 6557-6566. <https://doi.org/10.1039/C3AY41160E>

10. Pucher, Matthias, Urban Wünsch, Gabriele Weigelhofer, Kathleen Murphy, Thomas Hein, Daniel Graeber. 2019. “staRdom: versatile software for analyzing spectroscopic data of dissolved organic matter in R.” *Water* 11: 2366. <https://doi.org/10.3390/w11112366>

11. Stedmon, Colin A, Stiig Markager. 2005. “Tracing the production and degradation of autochthonous fractions of dissolved organic matter by fluorescence analysis.” *Limnology and Oceanography* 50: 1415-1426. <https://doi.org/10.4319/lo.2005.50.5.1415>

12. Gao, Zhiyuan, Céline Guéguen. 2017. “Size distribution of absorbing and fluorescing DOM in Beaufort Sea, Canada Basin.” *Deep Sea Research Part I: Oceanographic Research Papers* 121: 30-37. <https://doi.org/10.1016/j.dsr.2016.12.014>

13. Chen, Lin, Zaiyu Chen, Yuchen Wang, Yongkang Mao, Zongting Cai. 2021. “Effective treatment of leachate concentrate using membrane distillation coupled with electrochemical oxidation.” *Separation and Purification Technology* 267: 118679. <https://doi.org/10.1016/j.seppur.2021.118679>

14. Murphy, Kathleen R, Rasmus Bro, Colin A Stedmon. 2014. “Chemometric analysis of organic matter fluorescence.” *Aquatic organic matter fluorescence* 261: 339-375. <https://books.google.de/books?id=wTWNAwAAQBAJ&lpg=PA339&ots=s8aPykCc6i&dq=Chemometric%20analysis%20of%20organic%20matter%20fluorescence&lr&hl=zh-CN&pg=PA327#v=onepage&q&f=false>

15. Liu, Cheng, Yiheng Du, Hongbin Yin, Chengxin Fan, Kaining Chen, Jicheng Zhong, Xiaozhi Gu. 2019. “Exchanges of nitrogen and phosphorus across the sediment-water interface influenced by the external suspended particulate matter and the residual matter after dredging.” *Environmental Pollution* 246: 207-216. <https://doi.org/10.1016/j.envpol.2018.11.092>
